# Properties of tests for knee joint threshold to detect passive motion following anterior cruciate ligament injury: a systematic review and meta-analysis

**DOI:** 10.1186/s13018-022-03033-4

**Published:** 2022-03-04

**Authors:** Andrew Strong, Ashokan Arumugam, Eva Tengman, Ulrik Röijezon, Charlotte K. Häger

**Affiliations:** 1 Department of Community Medicine and Rehabilitation, Physiotherapy Section, Umeå University, Umeå, Sweden; 2 Department of Physiotherapy, College of Health Sciences, University of Sharjah, Sharjah, United Arab Emirates; 3 Department of Health, Learning and Technology, Physiotherapy Section, Luleå University of Technology, Luleå, Sweden

**Keywords:** Proprioception, Kinesthesia, Reliability, Validity, Responsiveness

## Abstract

**Background:**

Threshold to detect passive motion (TTDPM) tests of the knee joint are commonly implemented among individuals with anterior cruciate ligament (ACL) injury to assess proprioceptive acuity. Their psychometric properties (PMPs), i.e. reliability, validity and responsiveness, are however unclear. This systematic review aimed to establish the PMPs of existing knee joint TTDPM tests among individuals with ACL injury.

**Methods:**

The databases PubMed, AMED, CINAHL, SPORTDiscus, Web of Science, Scopus, CENTRAL and ProQuest were searched to identify studies that assessed the properties of knee joint TTDPM tests in individuals with ACL injury. The risk of bias for each included study was assessed at the outcome level for each test. Overall quality and levels of evidence for each property were rated according to established criteria. Meta-analyses with mean differences were conducted using random-effects models when adequate data were available.

**Results:**

Fifty-one studies covering 108 TTDPM tests and 1632 individuals with unilateral ACL injury were included. A moderate-to-strong level of evidence indicated insufficient quality for all of the following: convergent validity, known-groups validity, discriminative validity, responsiveness between subgroups, and responsiveness to intervention. Subgroup meta-analyses for known-groups validity did however find that a starting angle of 15° resulted in significantly worse TTDPM for knees with ACL injury compared to those of asymptomatic persons (mean difference 0.28°; 95% CI 0.03 to 0.53; *P* = 0.03), albeit based on only three studies. Due to the lack of evidence, it was not possible to estimate the quality of reliability, measurement error, and criterion validity, nor responsiveness from a criterion and construct approach.

**Conclusions:**

Among persons with ACL injury, existing tests of knee joint TTDPM lack either sufficient quality or evidence for their reliability, validity and responsiveness. Significantly worse thresholds for ACL-injured knees compared to those of asymptomatic controls from a 15° starting angle and trends towards significance for some validity measures nevertheless encourage the development of standardised tests. Further research investigating the influence of modifiable test components (e.g. starting angle and motion direction) on the PMPs of knee joint TTDPM tests following ACL injury is warranted.

**Supplementary Information:**

The online version contains supplementary material available at 10.1186/s13018-022-03033-4.

## Background

Rupture of the anterior cruciate ligament (ACL) is believed to cause alterations to proprioceptive acuity at the knee joint due to damage and loss of its receptors [1]. Afferent information from these receptors is ordinarily received and interpreted by the brain for an understanding of the applied forces, position and movement at the knee joint [2]. These different proprioceptive senses are subsequently assessed separately using common tests [3]. One common test used to assess sensibility to movement is threshold to detect passive motion (TTDPM). A typical TTDPM test involves a blindfolded individual indicating when they first detect passive motion at the joint. The outcome variable is most often the degrees that the joint has moved or alternatively the time that has elapsed before the participant signals the detection of motion. An additional dichotomous outcome is the indication of motion direction, e.g. flexion or extension. Previous meta-analyses have found significantly poorer TTDPM for knees with ACL injury compared to those of asymptomatic controls [4] (known-groups validity) and the contralateral non-injured side [4, 5] (operationally defined as discriminative validity). However, the respective authors included some participants in the same meta-analyses multiple times due to them performing multiple test setups in the same studies, potentially confounding the results. The reliability of the included tests was also not reported despite its importance regarding the consistency of the results. A review of the responsiveness of TTDPM tests at the knee after ACL injury is also entirely lacking.

A standardised TTDPM test with established PMPs would facilitate the assessment of knee proprioception among those with ACL injury to potentially identify individuals with such deficits. Identification could subsequently lead to targeted interventions to improve rehabilitation outcomes and potentially reduce the reported 30 to 40 times higher risk of secondary ACL injury [6] and 4 to 6 times higher odds of developing knee osteoarthritis [7]. A comprehensive and up-to-date consolidation of the existing literature regarding the PMPs of TTDPM tests could thus guide researchers and clinicians in the implementation and/or development of such tests. The current article forms part of a review series synthesising the PMPs of knee proprioception tests among individuals with ACL injury and asymptomatic persons [8]. The aim of this review was to assimilate the level of evidence for and quality of knee joint TTDPM tests among persons with ACL injury who have been treated with or without surgical reconstruction. We hypothesised that in line with previous meta-analyses, knee joint TTDPM tests would show sufficient known-groups and discriminative validity among individuals with ACL injury. We further hypothesised that remaining PMPs would either show insufficient quality or insufficient evidence to estimate their quality.

## Methods

In accordance with our published protocol [8], which was registered in the International Prospective Register of Systematic Reviews (PROSPERO; registration number CRD42018108014), the Preferred Reporting Items for Systematic Reviews and Meta-Analysis (PRISMA) guidelines [9, 10] were used to conduct and report this review.

### Eligibility criteria

Eligibility criteria for study inclusion and exclusion are provided in Table 1. To summarize, studies had to include participants ≥ 10 years of age with ACL injury, irrespective of treatment strategy, who had performed at least one test of knee joint TTDPM. At least one PMP (reliability, validity or responsiveness) relating to the outcome measure(s) of the TTDPM test(s) must have been investigated.

**Table 1:** Eligibility criteria for studies

Category	Details
*Inclusion criteria*	
Participants	Aged ≥ 10 years with anterior cruciate ligament injury managed with or without surgical reconstruction
Construct	At least one specific method of measuring knee joint threshold to detect passive motion
Equipment	Any equipment that is capable of quantifying knee joint threshold to detect passive motion
Setting	The test can be performed in any setting including a laboratory or a clinic
Outcome measures	Studies designed to investigate at least one of the following psychometric properties: reliability; measurement error; criterion validity (concurrent or predictive); hypothesis testing (convergent, known-groups or discriminative validity), and responsiveness
Study type	(1) The primary or sole aim of investigating at least one psychometric property of a knee joint threshold to detect passive motion test, (2) Reliability, validity or responsiveness reported as secondary or additional findings on the condition that sufficient details are included to rate the methodological quality/risk of bias, (3) Studies which have included data separately for individuals with anterior cruciate ligament injury, other lower-limb disorders and knee-healthy controls, (4) Peer-reviewed observational studies, cross-sectional studies, randomised controlled clinical trials or quasi-experimental studies
Language	English language only
Access	Full text publications retrievable via electronic database or manual search
*Exclusion criteria*	
Construct	(1) Validation of self-reported knee function and/or physical activity levels without addressing specific knee joint threshold to detect passive motion tests, and/or (2) Validation of proprioception-related function, such as knee joint position sense, not specifically assessing knee joint threshold to detect passive motion
Equipment	Validation of measurement instruments not specifically designed to assess knee joint threshold to detect passive motion
Outcome measures	Measures not addressing any psychometric properties of a knee joint threshold to detect passive motion
Study type	Pilot studies, abstracts, systematic reviews and meta-analyses, narrative reviews, book reviews, case series/reports, commentaries, editorials, letters to the editor, patient education handouts, consensus statements, clinical practice guidelines, theses/dissertations or unpublished literature

### Search strategy and study selection

One researcher (AS) performed a systematic search, as detailed in our published review protocol [8], in the following databases from their inception to 17 February 2021: PubMed; Allied and Complementary Medicine (AMED via EBSCO); the Cumulative Index to Nursing and Allied Health Literature ([CINAHL] via EBSCO); SPORTDiscus (via EBSCO); Web of Science; Scopus; the Cochrane Central Register of Controlled Trials (CENTRAL); Physical Education Index (via ProQuest). An additional search using relevant terms was performed in Google Scholar.

Due to our search encompassing all tests of proprioception as part of our review series, further details of the study selection process are provided in our previous review [11]. Briefly, after removal of duplicates, two authors (AS, ET) independently screened all titles, abstracts and full texts of relevance according to the stated eligibility criteria. References lists of the included studies were also screened for any additional studies of relevance.

### Data extraction and risk-of-bias assessment

Data required for risk of bias (RoB) assessment and analysis syntheses, e.g. testing procedures and results, were extracted from all eligible studies independently by two assigned research assistants (IA 50%, KO 50%) and verified by a second researcher (AS 50%, AA 50%). A third researcher was available in case of disagreements (ET). In cases of missing or inadequate data, contact was made with the study author(s) requesting the necessary information.

The COnsensus-based Standards for the selection of health Measurement INstruments (COSMIN) RoB checklist [12] was used to assess and rate the RoB of each outcome measure of each study. Methodological quality was therefore rated according to a four-point scale (“inadequate” (high risk of bias), “doubtful”, “adequate” and “very good” (low risk of bias)). After removing all outcome measures rated as “inadequate”, the following PMPs were assessed at the outcome level: reliability, measurement error, criterion (concurrent or predictive) validity, hypothesis testing for construct validity (convergent [comparison to other outcome measures], known-groups validity [operationally defined as ACL-injured knees compared to either asymptomatic knees of other persons or ACL-injured knees of otherwise asymptomatic individuals]), discriminative validity [operationally defined as ACL-injured knees compared to the contralateral asymptomatic knees of the same individuals], and responsiveness (criterion and construct approach). Data were then extracted independently by an assigned research assistant (IA 50%, KO 50%) and verified by a second researcher (AS 50%, AA 50%). A third researcher (ET) was available in case of disagreements, but all matters were resolved through consensus.

Within studies, TTDPM tests were considered independent when results were presented separately and at least one modifiable component, e.g. starting knee angle, differed. When an ACL group underwent reconstructive surgery during the study and measurements were performed pre- and post-reconstruction, they were classified as ACL-reconstructed (ACLR) rather than ACL-deficient (ACLD). When participants were assessed at multiple time points and assessed for known-groups validity or discriminative validity, data for only the last test were extracted.

### Quality assessment of psychometric properties

To assess the quality of each PMP, we used criteria adapted from Prinsen et al. [13] (Additional file 1: Table S1). Briefly, possible ratings were “sufficient”, “indeterminate” or “insufficient”, depending on whether the synthesised study outcomes were in agreement with the threshold cut-off values of relevant statistical parameters or hypotheses. For hypotheses testing for construct validity and responsiveness, ratings of “sufficient” and “insufficient” required 75% of the findings to favour the hypothesis (positive) or not favour the hypothesis (negative), respectively. If less than 75% of the findings were in agreement, the quality of the PMP was rated as “indeterminate” due to the conflicting level of evidence. A quality rating of “not estimable” was given when there was an unknown level of evidence, i.e. when a PMP was either assessed only in studies with an RoB rating of “inadequate” or not investigated at all.

### Level of evidence

To assess the level of evidence for each PMP, we used a scale adapted by Kroman and colleagues [14] (Additional file 2: Table S2). Rating criteria were as follows: *strong*—evaluated in multiple studies with an RoB rating of adequate or at least one study with an RoB rating of very good (i.e. a low RoB); *moderate*—evaluated in multiple studies with an RoB rating of doubtful or at least one study with an RoB rating of adequate; *limited*—evaluated in one study with an RoB rating of doubtful; *conflicting*—evaluated in studies with contradictory findings; *unknown*—evaluated only in studies with and RoB rating of inadequate or not investigated at all.

### Meta-analysis

Meta-analyses were performed in the Review Manager (RevMan) computer program (version 5.3. Copenhagen: The Nordic Cochrane Centre, The Cochrane Collaboration, 2014) when adequate (homogenous) data were available from at least three studies with an RoB rating of “doubtful”, “adequate” or “very good”. When groups from a study were included for multiple comparisons in the same meta-analysis, their totals were divided by the number of comparisons for which they were included to better reflect their weighting on the findings, e.g. Nagai (2013) reported results for their TTDPM test separately for flexion and extension for the same ACL group of 22 individuals and thus the number of participants for the two separate comparisons was halved to 11 each. A random-effects model was applied due to differences in the way the studies were conducted and to consider sampling error [15]. The mean difference (MD) was used because all relevant outcome measures were provided in the same unit (degrees) [16]. When only median values with measures of dispersion were reported in the studies, means and standard deviations were estimated based on the method by Wan and colleagues [17]. Statistical heterogeneity was considered present if the *I*^2^ statistic exceeded 40% [18]. Funnel plots to help identify for the presence of publication bias were considered appropriate only when at least ten studies were included in the meta-analysis [19]. Subgroups were created within each meta-analysis according to ACLR or ACLD knees to investigate the potential influence of treatment strategy. Sensitivity analyses were performed for methodological quality and test procedure by restricting the meta-analyses to studies with an RoB rating of “adequate” or “very good” and specific starting knee angles, respectively. Statistical significance was set at *P* < 0.05.

For known-groups validity, data pertaining to the non-dominant leg of the control group were used for a more stringent comparison to the ACL groups. In cases when side dominance was not indicated, data for the left leg of controls were extracted to increase the likelihood of selecting the non-dominant leg. For responsiveness, baseline data were compared only to data collected at the final follow-up in the respective study.

## Results

### Search results

**Fig. 1:**
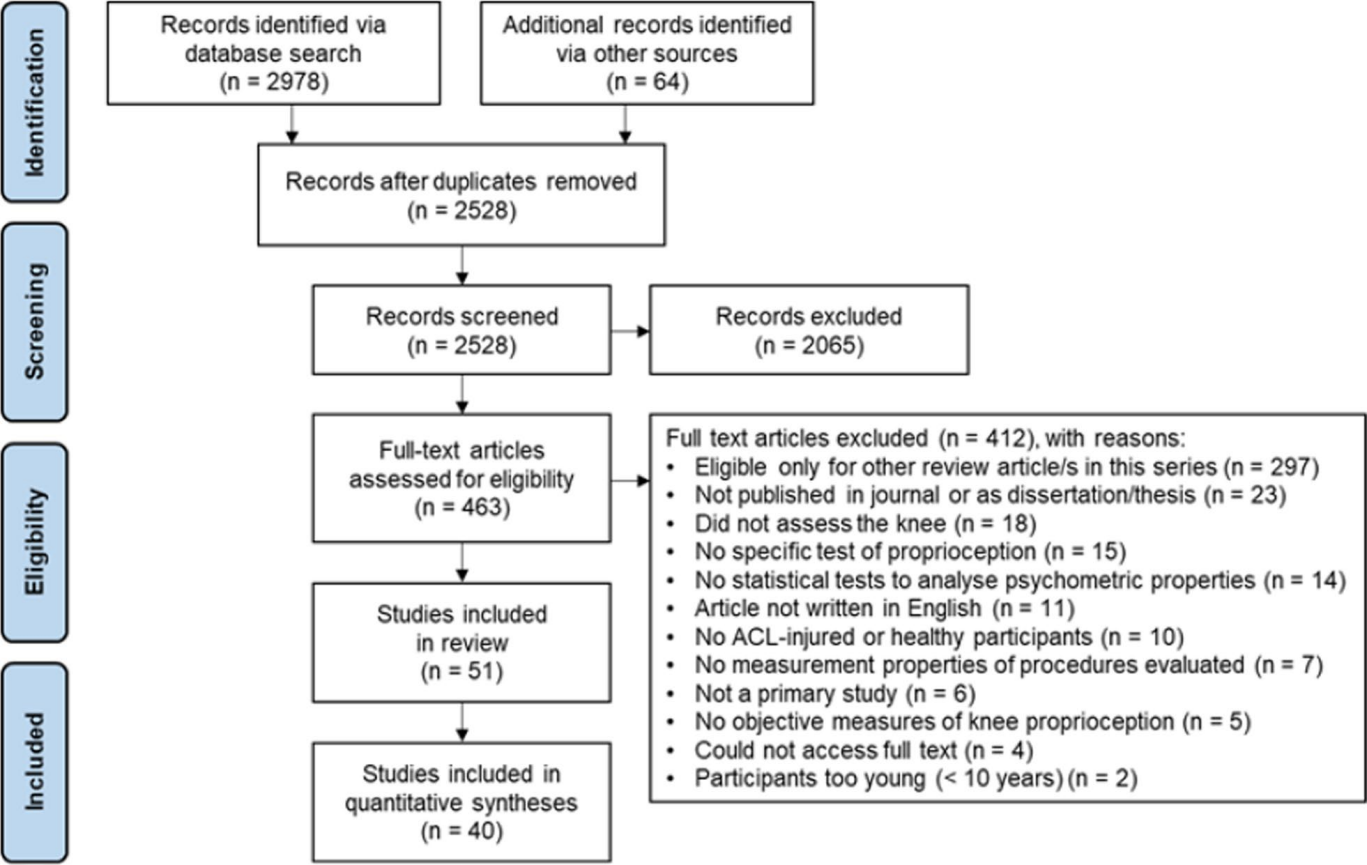
PRISMA flow diagram illustrating the search process and results for studies involving TTDPM of the knee

Full search results are illustrated in Fig. 1. Briefly, the systematic electronic database search identified 2978 studies and a further 64 were retrieved via manual searches. Following duplicate removal, the titles and abstracts of 2528 studies were screened. The full texts of 463 of these studies were then screened for eligibility, and 51 studies were subsequently included in this review.

### Characteristics of the included studies

Characteristics of all included studies are detailed in Additional file 3: Table S3. In summary, across the 51 studies, we identified 1632 participants with a unilateral ACL injury from 75 different cohorts. Of these cohorts, 43 were ACLR and 32 were ACLD. Sex distribution was reported for 70 (93%) of the ACL populations, of which 45% were females. The mean weighted age of the ACL participants, based on 74 ACL populations for which mean age was reported, was 27.9 years. A total of 108 different knee joint TTDPM tests were identified across the 51 studies. The most common direction of motion was towards extension (40 tests), followed by flexion (38 tests); a combination of extension and flexion was used in 28 studies and two studies did not report motion direction. The most common equipment used was a motor pulley (18 studies), most common body position was sitting (29 studies), and most common angular velocity was 0.5°/s (28 studies). The most common starting knee angles were 45° (30 tests), 15° (28 tests), 20° (21 tests) and 40° (19 tests) knee flexion; multiple angles were used for 20 tests.

### Risk of bias assessment

Assessment of RoB was performed at the outcome level rather than for each study as a whole to provide a more accurate assessment of each PMP. For example, across the PMPs of discriminative and known-groups validity, one study [20] had investigated both properties but did not provide information regarding matching of activity level between groups and thus the rating was marked down for known-groups validity but not for discriminative validity. Also, for convergent validity (i.e. the degree to which the outcomes are similar to those of other outcome measures), three studies [21–23] assessed correlations to other measurement instruments which were considered reliable and valid for the population of study, but also subjective function for which evidence for these PMPs was lacking. Results of the RoB assessment are provided in Additional file 4: Table S4.

### Assessment of quality and level of evidence of the included studies

#### Reliability and measurement error

No studies evaluated reliability. One study [22] assessed measurement error, but this received an RoB rating of “inadequate”. The quality of reliability and measurement error was therefore not estimable due to the unknown level of evidence.

#### Criterion validity

No studies evaluated criterion validity, and thus, its quality was not estimable due to the unknown level of evidence.

#### Convergent validity

Convergent validity was assessed for 50 different outcome measures across 20 studies (Additional file 5: Table S5). Three studies [21–23] which assessed multiple outcomes received RoB ratings of “doubtful” for each outcome with the exception of subjective function which was rated “inadequate” for each study. For the remaining 17 studies, the same RoB rating was given even when multiple outcome measures were assessed and was “doubtful” for 11 [24–34] and “inadequate” for six [35–40] studies. The most commonly assessed outcome measures were knee joint laxity (six studies), single-leg hop distance, gender, current Tegner activity scale score, and subjective evaluation (all four studies each). Meta-analyses for the respective outcome measures were however not performed due to either studies with an RoB rating of “inadequate” or a lack of reported data. Of the outcome measures which were assessed in three or more separate studies, none were significantly correlated in more than half of the results. Therefore, based on an overall moderate level of evidence, the convergent validity of TTDPM tests after ACL injury was found to be insufficient.

### Hypothesis testing

#### Known-groups validity

Known-groups validity was assessed in 96 group comparisons across 31 studies (Additional file 6: Table S6), two of which received an RoB rating of “very good” [41, 42], two “adequate” [26, 28], 12 “doubtful” [31, 33, 34, 38, 43–50] and 15 “inadequate” [20, 28, 29, 36, 40, 51–60]. Results from the related meta-analyses are presented henceforth.

##### ACL-injured knees versus asymptomatic controls

**Fig. 2:**
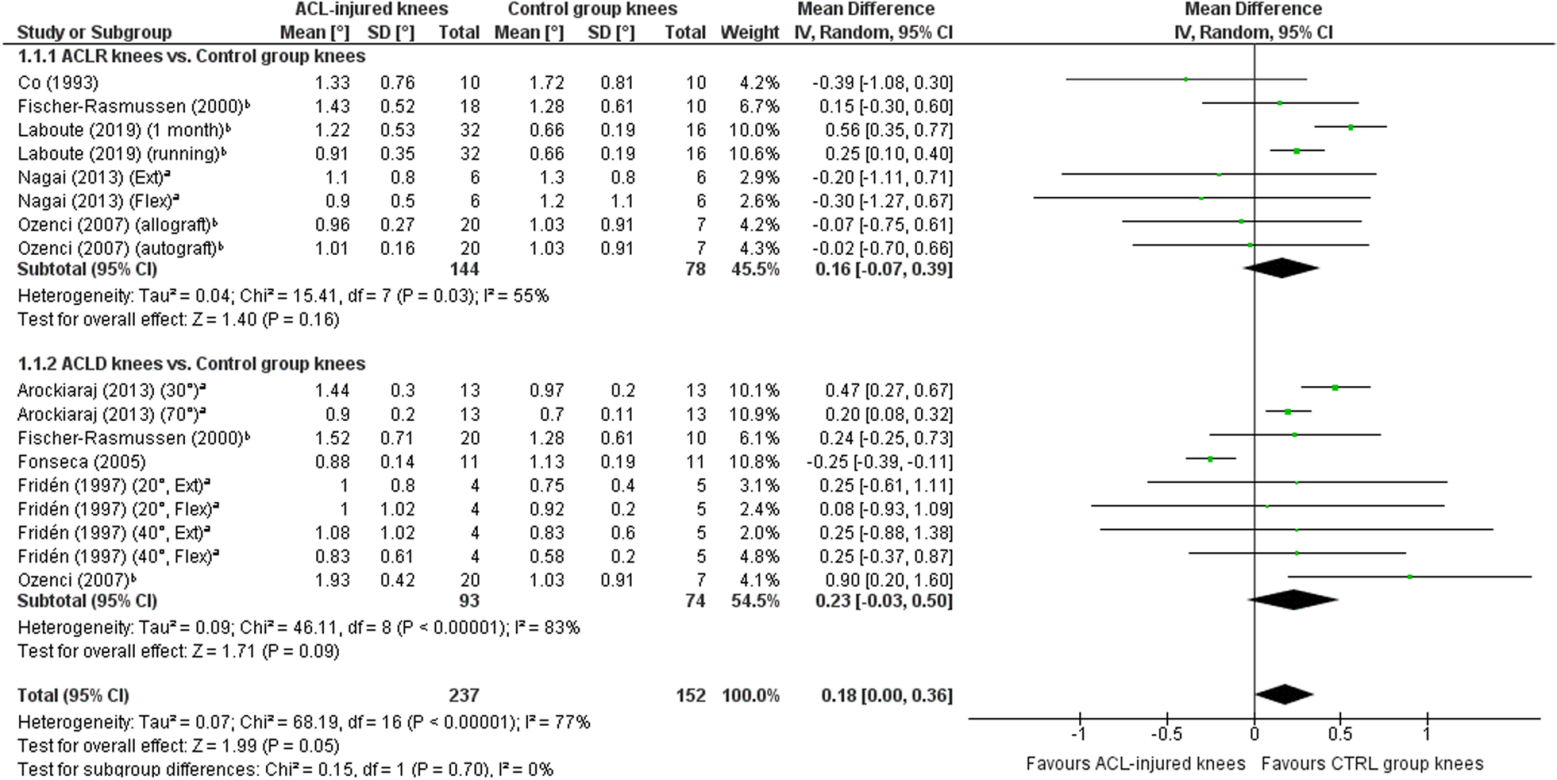
Forest plot of all studies included in the meta-analysis for known-groups validity. ^a^Group numbers (i.e. “Total”) for both groups have been divided by the number of times each group has been included in the meta-analysis. ^b^Group number (i.e. “Total”) for the control group only has been divided by the number of times the group has been included in the meta-analysis

**Fig. 3:**
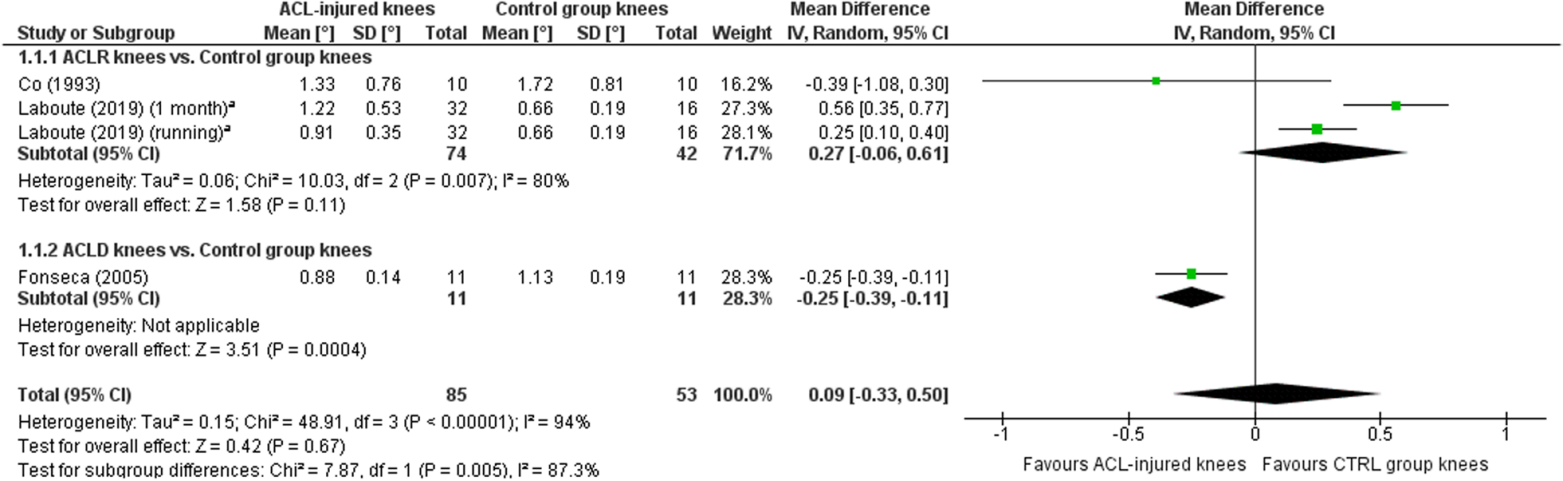
Forest plot of only those studies with a risk of bias rating of “adequate” or “very good” included in the meta-analysis for known-groups validity. ^a^Group number (i.e. “Total”) for the control group only has been divided by the number of times the group has been included in the meta-analysis

**Fig. 4:**
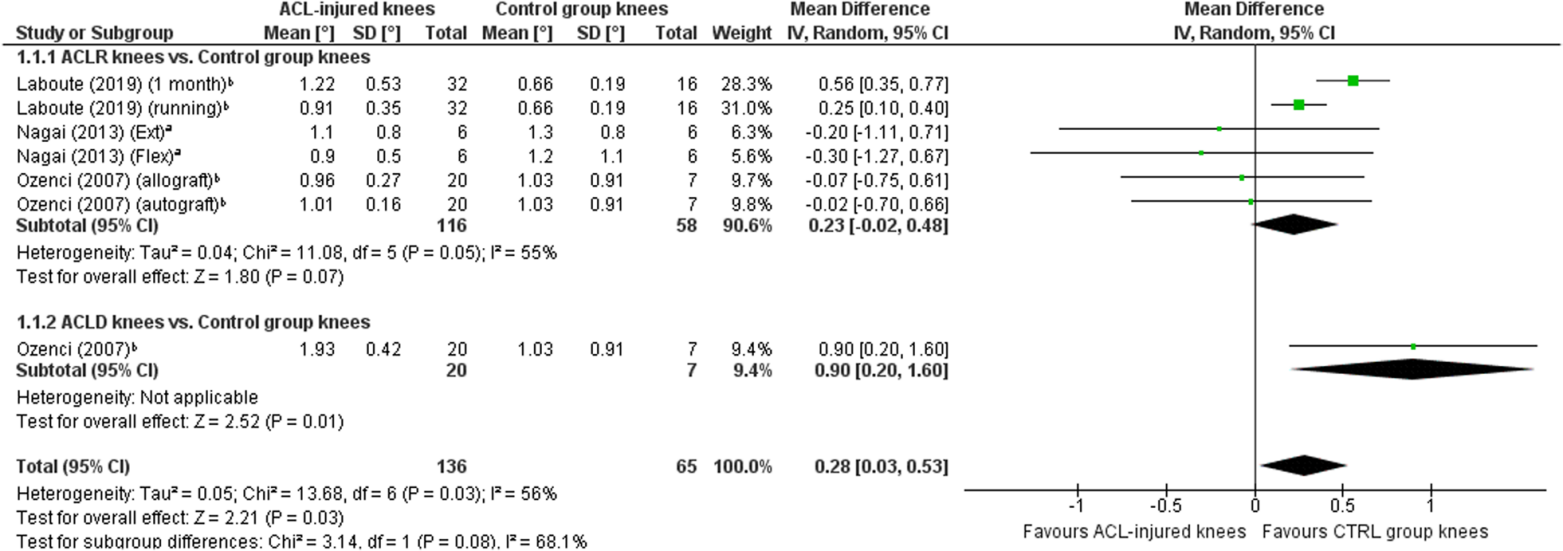
Forest plot of the studies included in the main meta-analysis for known-groups validity which used 15° as the starting angle. ^a^Group numbers (i.e. “Total”) for both groups have been divided by the number of times each group has been included in the meta-analysis. ^b^Group number (i.e. “Total”) for the control group only has been divided by the number of times the group has been included in the meta-analysis

A meta-analysis which included 17 comparisons across eight studies [26, 31, 38, 41–44, 46] found a near-statistically significant trend towards worse TTDPM for knees with ACL injury compared with those of asymptomatic controls (MD 0.18°; 95% CI 0.00 to 0.36; *P* = 0.05; *I*^2^ = 77%) (Fig. 2). A sensitivity meta-analysis for methodological quality, which included four comparisons across three studies [26, 41, 42], found no significant difference between knees with ACL injury and those of asymptomatic controls (MD 0.09°; 95% CI − 0.33 to 0.50; *P* = 0.67; *I*^2^ = 94%) (Fig. 3). A sensitivity meta-analysis for starting angle was possible only for 15°, for which seven comparisons across three studies [38, 42, 46] found significantly poorer TTDPM for knees with ACL injury compared to those of asymptomatic controls (MD 0.28°; 95% CI 0.03 to 0.53; *P* = 0.03; *I*^2^ = 56%) (Fig. 4).

##### ACLR knees versus asymptomatic controls

**Fig. 5:**
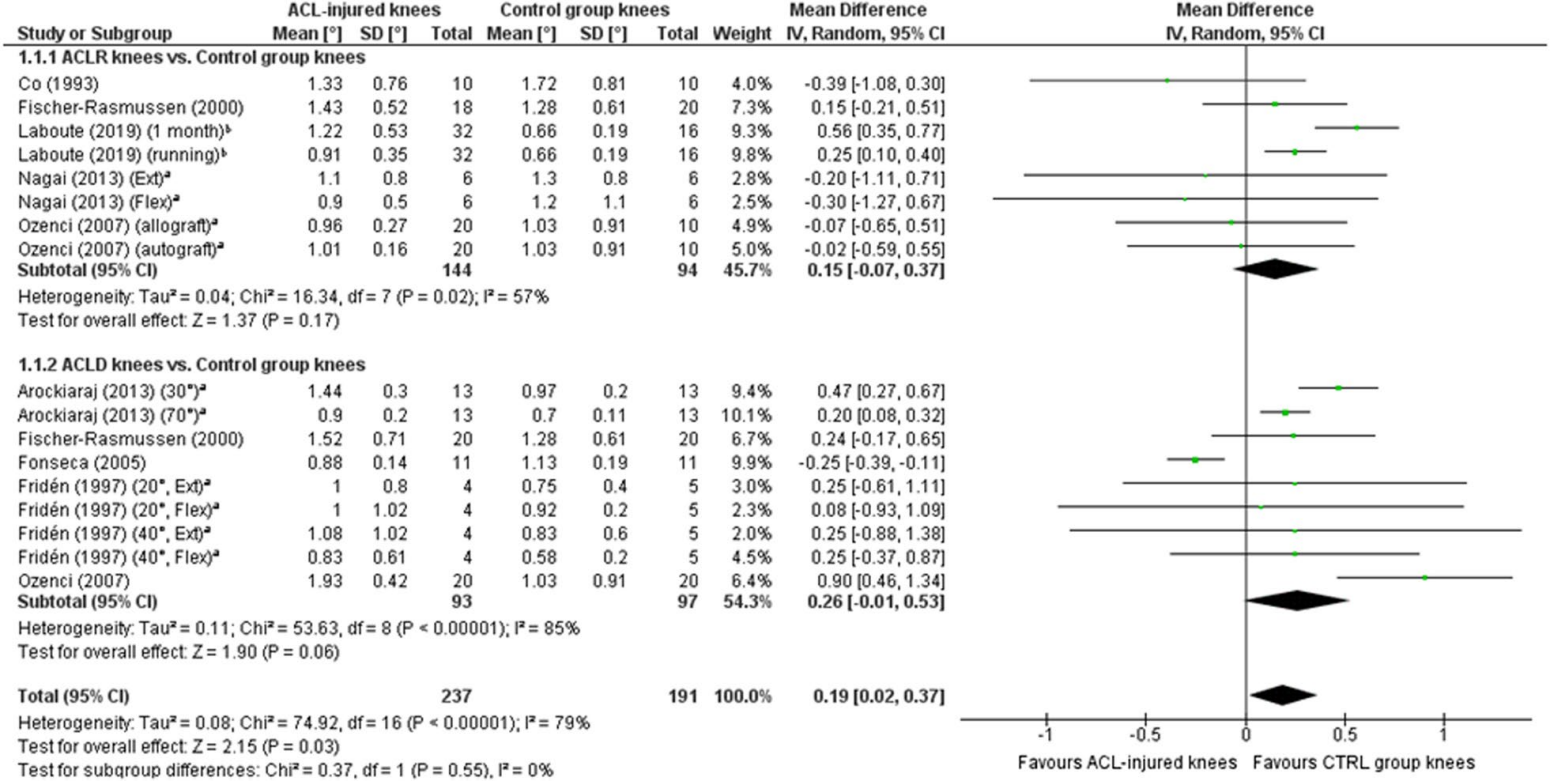
Forest plot of all studies included in the subgroup meta-analyses for known-groups validity. ^a^Group numbers (i.e. “Total”) for both groups have been divided by the number of times each group has been included in the specific subgroup meta-analysis. ^b^Group number (i.e. “Total”) for the control group only has been divided by the number of times the group has been included in the specific subgroup meta-analysis

Eight comparisons across five studies [26, 31, 38, 42, 46] were included in a meta-analysis which found no significant difference between knees (MD 0.16°; 95% CI − 0.07 to 0.39; *P* = 0.16; *I*^2^ = 55%) (Fig. 5).

##### ACLD knees versus asymptomatic controls

Nine comparisons across five studies [31, 38, 41, 43, 44] were included in a meta-analysis which found no significant difference between knees (MD 0.23°; 95% CI − 0.03 to 0.50; *P* = 0.09; *I*^2^ = 83%) (Fig. 5).

##### Overall quality of known-groups validity and level of evidence

Strong evidence indicates insufficient quality for known-groups validity across knee joint TTDPM tests when including all studies and when only including those in higher quality (low RoB) studies. Sufficient validity was however found for those tests with a 15° starting angle, albeit from only three studies.

#### Discriminative validity

Discriminative validity was assessed in 75 between-leg comparisons across 30 studies (Additional file 7: Table S7), two of which received an RoB rating of “very good” [41, 42], two “adequate” [26, 28], 12 “doubtful” [20, 24, 31–33, 38, 44, 46, 48, 59, 61] and 14 “inadequate” [25, 34, 35, 39, 43, 51, 54–58, 62–64]. Results from the meta-analyses are presented henceforth.

##### ACL-injured versus contralateral non-injured knees

**Fig. 6:**
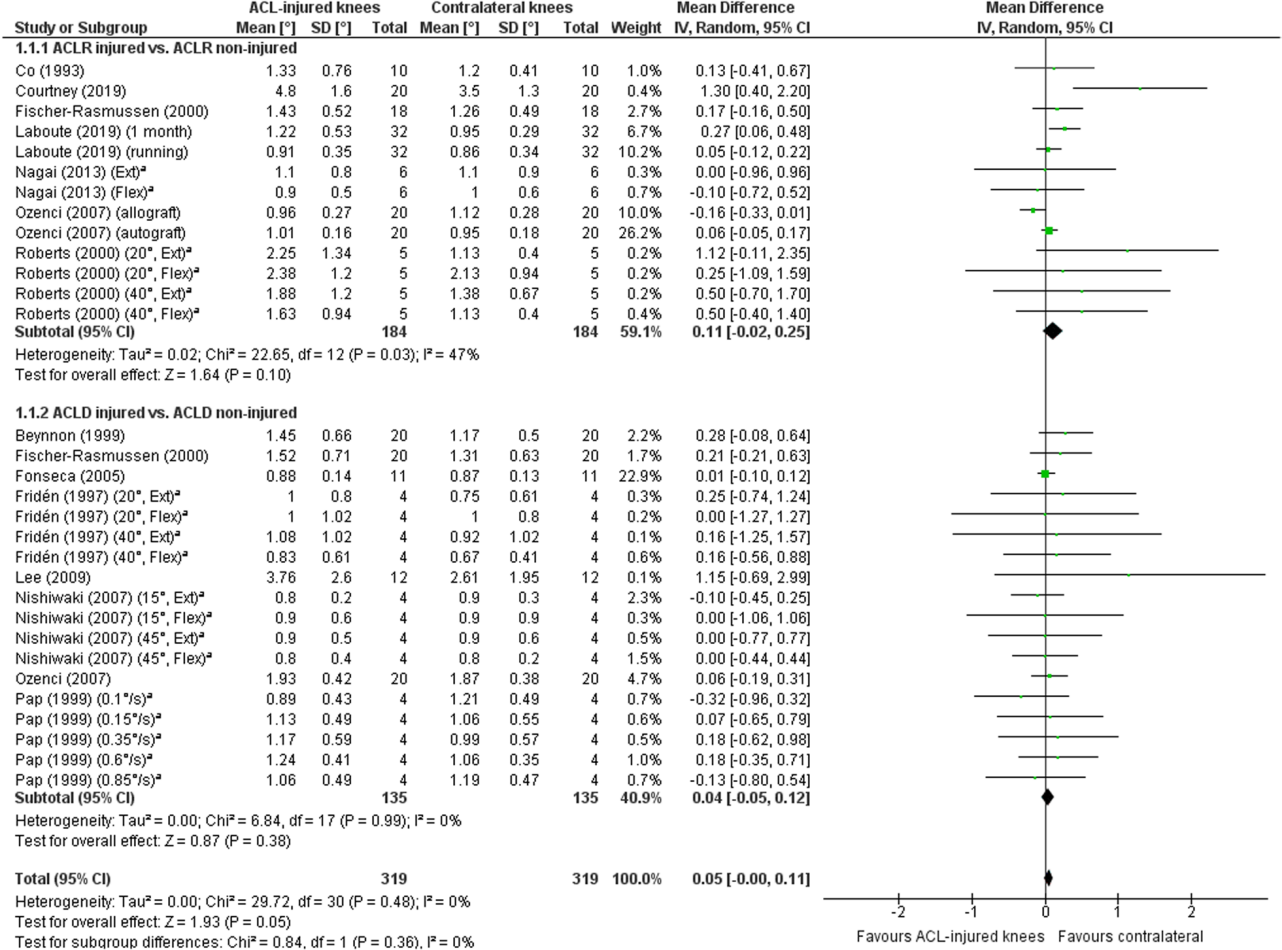
Forest plot of all studies included in the meta-analysis for discriminative validity. ^a^Group numbers (i.e. “Total”) have been divided by the number of times each group has been included in the meta-analysis

**Fig. 7:**
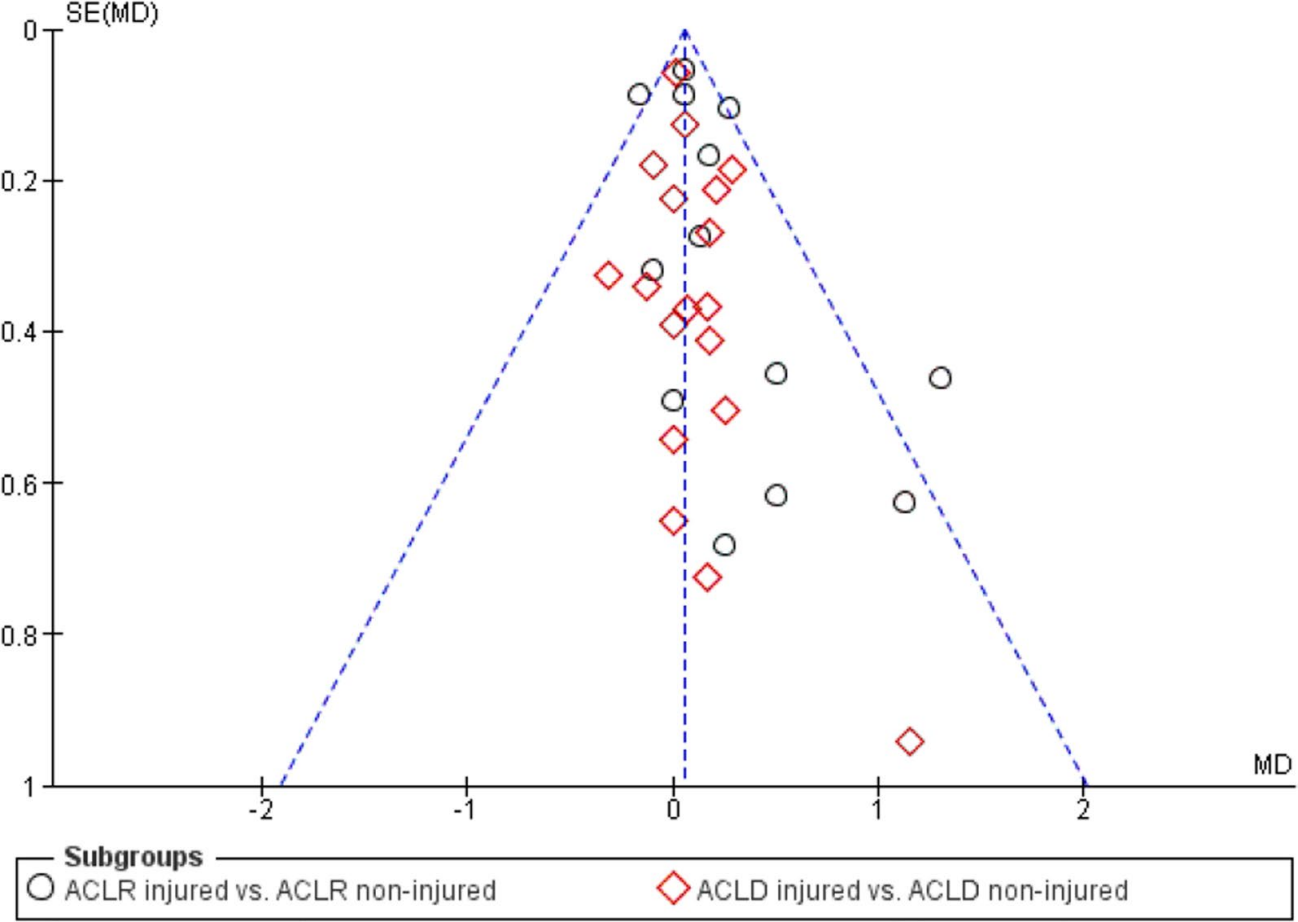
Funnel plot of all studies included in the meta-analysis for discriminative validity. Abbreviations: ACLD, anterior cruciate ligament-deficient; ACLR, anterior cruciate ligament-reconstructed; MD, mean difference; SE, standard error

**Fig. 8:**
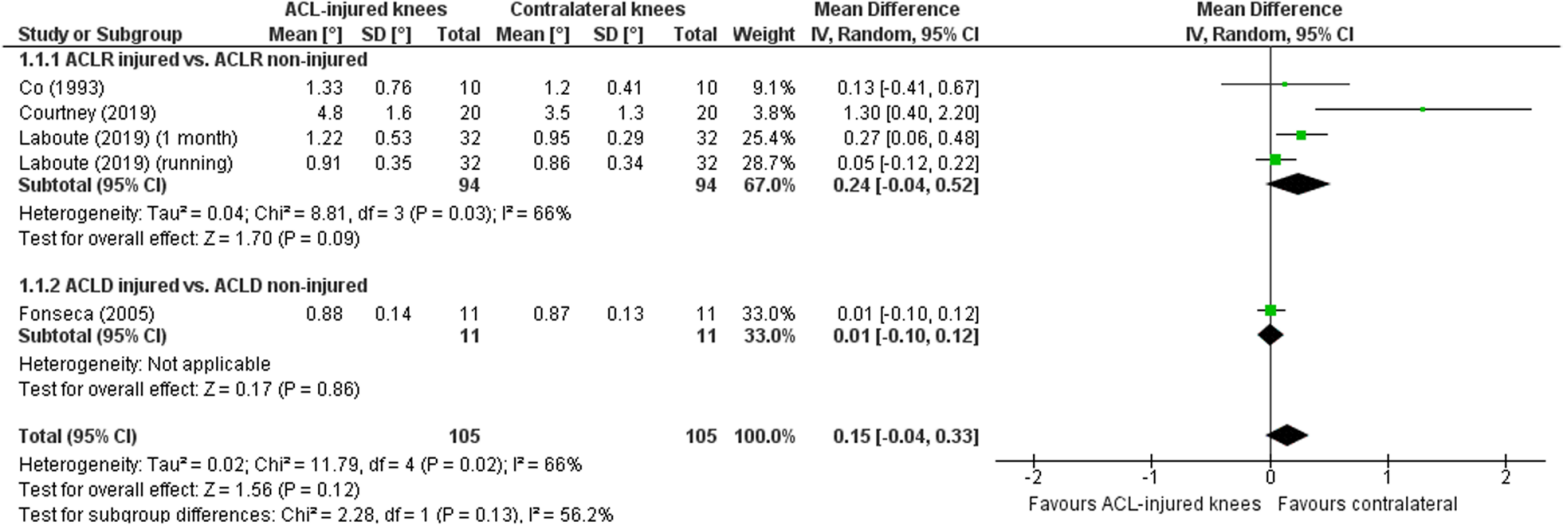
Forest plot of only those studies with a risk of bias rating of “adequate” or “very good” included in the meta-analysis for discriminative validity

**Fig. 9:**
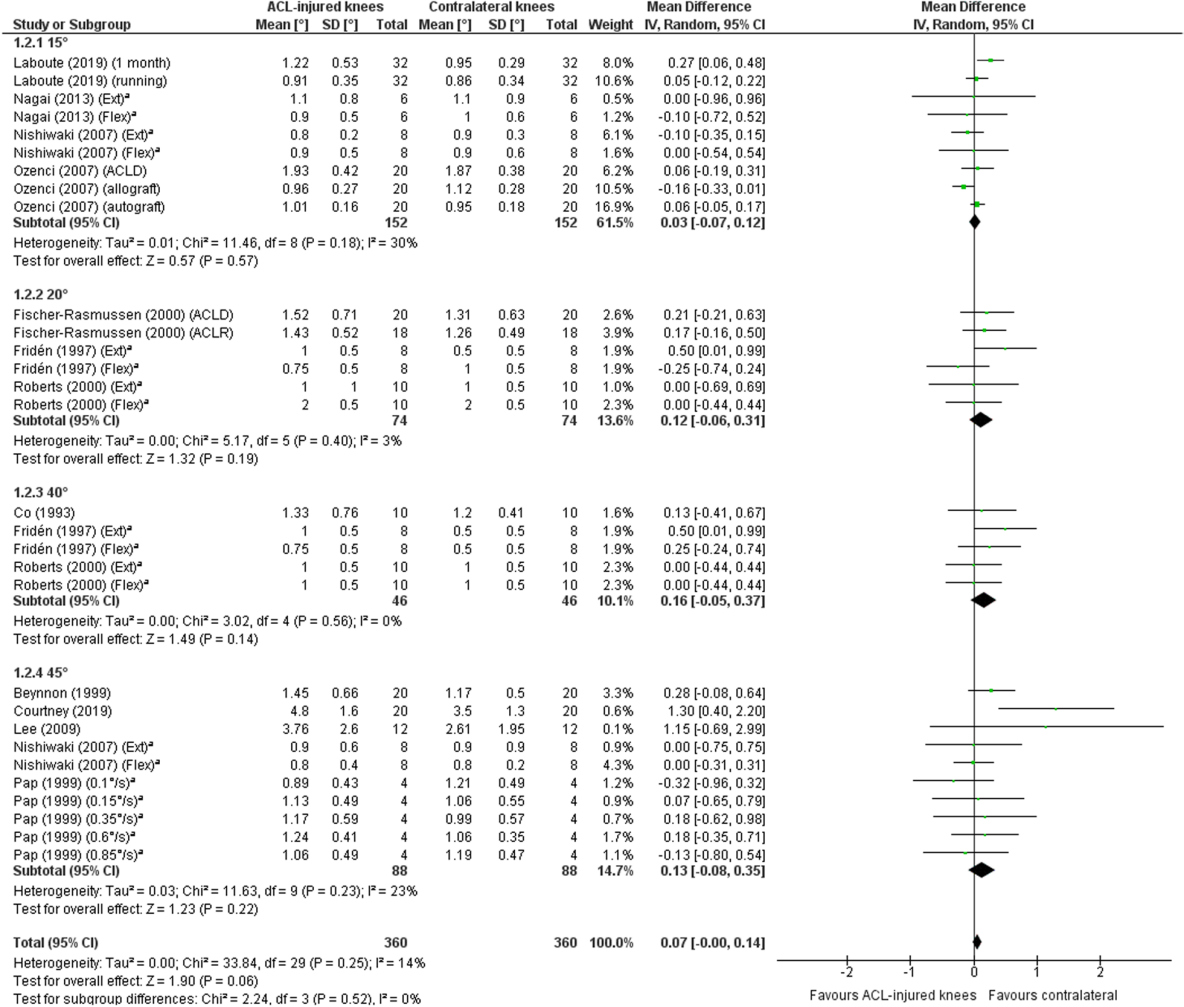
Forest plot of the studies included in the main meta-analysis for discriminative validity as subgroups depending on the starting knee angle used in the test (15°, 20°, 40° or 45°). One study [41] was omitted because it was the only one to use 35° as the starting angle. ^a^Group numbers (i.e. “Total”) have been divided by the number of times each group has been included in the respective subgroup meta-analysis

A meta-analysis which included 31 comparisons across 13 studies [20, 24, 26, 28, 31, 32, 37, 38, 41, 42, 44, 46, 59] found poorer TTDPM for ACL-injured knees compared to the contralateral non-injured knees close to statistical significance (MD 0.05°; 95% CI − 0.00 to 0.11; *P* = 0.05; *I*^2^ = 0%) (Fig. 6). A funnel plot (Fig. 7) indicated potential publication bias due to asymmetry. A sensitivity meta-analysis for methodological quality, which included five comparisons across four studies [26, 28, 41, 42], found no significant difference between knees (MD 0.15°; 95% CI − 0.04 to 0.33; *P* = 0.12; *I*^2^ = 66%) (Fig. 8). Subgroup meta-analyses found no significant difference between knees for starting angles of 15° (nine comparisons, four studies [32, 38, 42, 46]: MD 0.03°; 95% CI − 0.07 to 0.12; *P* = 0.57; *I*^2^ = 30%), 20° (six comparisons, three studies [31, 44, 59]: MD 0.12°; 95% CI − 0.06 to 0.31; *P* = 0.19; *I*^2^ = 3%), 40° (five comparisons, three studies [26, 44, 59]: MD 0.16°; 95% CI − 0.05 to 0.37; *P* = 0.14; *I*^2^ = 0%) and 45° (ten comparisons, five studies [20, 24, 28, 32, 37]: MD 0.13°; 95% CI − 0.08 to 0.35; *P* = 0.22; *I*^2^ = 23%) (Fig. 9).

##### ACLR versus contralateral non-injured knees

Thirteen comparisons across seven studies [26, 28, 31, 38, 42, 46, 59] were included in a meta-analysis which found no significant difference between knees (MD 0.11°; 95% CI − 0.02 to 0.25; *P* = 0.10; *I*^2^ = 47%) (Fig. 6).

##### ACLD versus contralateral non-injured knees

Eighteen comparisons across eight studies [20, 24, 31, 32, 37, 38, 41, 44] were included in a meta-analysis which found no significant difference between knees (MD 0.04°; 95% CI − 0.05 to 0.12; *P* = 0.38; *I*^2^ = 0%) (Fig. 6).

##### Overall quality of discriminative validity and level of evidence

Strong evidence indicates insufficient quality for the discriminative validity of existing TTDPM tests, a finding which was unaffected by study quality (RoB) or starting angle.

### Responsiveness

#### Criterion approach and construct approach between other outcome measurements

No studies assessed responsiveness from a criterion approach nor between other outcome measurements, and thus, the unknown level of evidence meant that their quality was not estimable.

#### Construct approach between subgroups

Responsiveness between subgroups was assessed in seven studies (Additional file 8: Table S8), three of which received an RoB rating of “doubtful” [49, 50, 65] and the remaining four of “inadequate” [45, 51, 52, 66]. None of the studies with an RoB rating of “doubtful” found a significant difference in change scores between subgroups. Thus, a moderate level of evidence indicates insufficient quality of responsiveness between subgroups for knee joint TTDPM tests among individuals with ACL injury.

#### Construct approach before and after intervention

Responsiveness to intervention was assessed in ten studies [22, 24, 34, 50, 57, 61, 64, 65, 67, 68] (Additional file 9: Table S9). One study received an RoB rating of “very good” [68], one “adequate” [34], three “doubtful” [24, 50, 61] and five “inadequate” [22, 57, 64, 65, 67]. The study with an RoB rating of “very good” found no significant improvement in TTDPM two years after ACLR for one group with an accelerated and one with a non-accelerated rehabilitation programme. Across the four studies with a doubtful or adequate RoB rating, 12 before and after comparisons were investigated, ten of which found non-significant differences. The interventions, which were applied at the knee, were bracing, a neoprene sleeve and stochastic resonance. The one study that compared before and 12 months after surgical reconstruction made eight comparisons among the same ACL group depending on starting angle (15° or 45°), motion direction (flexion or extension) and angular velocity (0.1°/s or 0.2°/s) of the TTDPM test and found only significant improvement for two conditions (45° towards flexion and extension, both at 0.2°/s). Overall, a strong level of evidence therefore indicates insufficient quality for responsiveness to intervention of TTDPM tests among individuals with ACL injury.

## Discussion

The aim of this systematic review was to summarize the current evidence for and quality of the PMPs of TTDPM tests of the knee joint among individuals with an ACL injury. There was overall strong evidence to substantiate insufficient quality of known-groups validity (ACLR/ACLD knees vs. asymptomatic controls) and discriminative validity (ACLR/ACLD knees vs. contralateral non-injured knees of the same individuals) for knee joint TTDPM tests. Tests with a starting angle of 15º did however result in significantly poorer thresholds for ACL-injured knees compared to asymptomatic controls, although this finding was based on results from only three studies. The quality of reliability and criterion validity of knee joint TTDPM tests was not estimable owing to the lack of studies investigating these properties. The convergent validity (with other objective and subjective outcome measures) and responsiveness (between subgroups or following interventions) of TTDPM tests were deemed insufficient based on a moderate level of evidence.

### Reliability and measurement error

Remarkably enough, reliability was not analysed in any of the 51 included studies. The quality of this PMP is of particular importance for outcomes relating to responsiveness to intervention/subgroups and should therefore be established. Although one study by Roberts et al. [22] reported the standard error of measurement (SEM; 0.003°to 0.24°) for their TTDPM outcomes, the overall RoB was rated “inadequate” (Additional file 4: Table S4) because the study did not report whether the patients were stable between measurements (test–retest interval was one month). In our previous systematic review on the PMPs of knee joint position sense (JPS) tests [11] only three studies (with RoB ratings of “inadequate”) reported test–retest reliability and one study (with an RoB rating of “very good”) reported insufficient quality for intra-session reliability. In agreement with the current findings of the TTDPM tests, there is therefore a lack of evidence for the reliability of tests that attempt to assess kinesthesia, i.e. the proprioceptive senses of limb position and motion [69], of the knee joint among individuals with ACL injury.

### Criterion and convergent validity

No studies assessed criterion validity of knee joint TTDPM tests. Due to a lack of gold standard test to compare TTDPM tests, the PMP remains uncertain; nevertheless, future studies on concurrent and predictive validity of TTDPM tests are warranted in individuals with an ACL injury. Convergent validity was difficult to ascertain because TTDPM test outcomes were compared to 50 different outcome measures (knee laxity, self-reported outcomes [KOOS, Lysholm scale, Tegner scale, etc.], neuromotor control/functional tests, thigh muscle strength, etc.) (Additional file 5: Table S5). The most common outcome measure compared was knee laxity [24, 31, 33, 35, 39]. The quality of this PMP was insufficient because 83% of the reported results in all studies with an RoB rating better than “inadequate” revealed no significant correlations between knee joint TTDPM tests and other outcome measures. It must be noted that some of the constructs compared could be considered multi-factorial (e.g. self-reported outcomes), thereby leading to low correlations with TTDPM outcomes. Further research is however needed in this area to substantiate the convergent validity of TTDPM tests with other related constructs in individuals with ACL injury.

### Known-groups validity

An overall meta-analysis for known-groups validity revealed a near-significant trend towards favourable outcomes for knees of asymptomatic controls (MD, 0.18°; *P* = 0.05). This trend lends some support to a previous meta-analysis by Relph and Herrington [4] who found significantly poorer TTDPM for ACL-injured knees compared with those of controls (MD, 0.38°; *P* = 0.03). Their results were however based on only two studies which were also included in the current review. A more recent systematic review without meta-analysis conversely concluded from seven studies that TTDPM tests did not tend to find significant differences between ACL-injured knees and those of controls [70]. Our sensitivity meta-analysis for methodological quality included four studies and also did not find a significant difference. Based on the most recent and strongest evidence, it must therefore be concluded that existing tests of TTDPM have overall insufficient quality for known-groups validity. Our subgroup meta-analysis did however reveal that a starting angle of 15º detected significantly poorer thresholds (MD, 0.28º, *P* = 0.03) for ACL-injured knees than those of asymptomatic controls, albeit based on outcomes from only three studies. This finding indicates that the choice of starting angle could be of particular importance for such comparisons and may help to guide future development of optimised TTDPM tests, for which the influence of angular velocity and motion direction should also be considered. Indeed, our previous review of knee JPS tests after ACL injury [11] found that test procedures, such as whether movements were passive or active, influenced the significance level of the results for known-groups validity.

### Discriminative validity

A meta-analysis for discriminative validity (Fig. 6) showed no significant difference in TTDPM for ACL-injured knees (ACLD/ACLR) compared to non-injured contralateral knees of the same individuals, although there was a trend towards statistical significance (MD, 0.05°; *P* = 0.05). There have been conflicting findings between previous systematic reviews [4, 5] to substantiate poorer TTDPM in ACL-injured knees compared to contralateral non-injured knees. The magnitude of proprioception loss following ACL injury appears to be more evident from tests of JPS than TTDPM [5]. Our own previous meta-analysis for knee JPS tests [11] further found that ACLD but not ACLR knees showed significantly poorer proprioception than the contralateral healthy knee, yet significant differences for TTDPM were not found for either group in the current meta-analysis. It must be noted that the test procedures differed between the studies in this review with regard to, e.g. angular velocities, direction of motion (flexion/extension), test device used and starting angle chosen. If a standardised test method were to be developed and applied consistently, then outcomes would be easier to interpret and potential differences between injured and non-injured knees may become apparent.

### Responsiveness

No study assessed responsiveness of TTDPM compared to a gold standard or other outcome measurements. A moderate and strong level of evidence was however found to establish insufficient quality of responsiveness between subgroups (ACLD/ACLR/asymptomatic controls) and following an intervention over a period of time, respectively. The interventions employed across the included studies were however diverse, e.g. knee brace, neoprene sleeve, neuromuscular training such as backward treadmill walking, etc., as were the study participants. Thus, due to the different interventions, populations and test setups, it is difficult to draw a general conclusion about responsiveness of knee joint TTDPM tests to interventions following ACL injury. A similar finding of a lack of significant result from diverse interventions was evident in our review of JPS tests after ACL injury [11]. It is possible that the included tests of knee TTDPM were not sensitive enough to identify change over time, or that kinesthesia may not be affected following ACL injury, or simply that the applied interventions simply did not influence knee proprioception.

### Methodological considerations

We did not include unpublished studies, gray literature or non-English studies which could be seen as a limitation. It remains unclear whether publication bias exists regarding TTDPM tests, but this can potentially lead to good studies being rejected because they do not present significant results. A funnel plot to assess for this in our meta-analyses was appropriate only for discriminative validity due to an otherwise lack of included studies. This funnel plot did however indicate a potential publication bias which may have affected our results and should be considered in future studies.

The completeness of descriptions regarding test procedures varied between the studies and, in addition, there were many different test procedures. It was therefore difficult to interpret whether one particular method was better than others in discerning changes in TTDPM tests following interventions or between groups. If these TTDPM tests should be used in future studies, a standardised method for testing is warranted. The participants across the included studies also represented a large range in terms of their quantity, age, sex and time since injury. One potentially important distinction in this context may also be that of non-copers/copers and adapters, i.e. those who do/do not experience giving way during daily activities and those who have adapted their activities to avoid giving way, respectively. Evidence suggests differences between such groups regarding TTDPM and somatosensory evoked potentials [62]. Any future research that attempts to develop knee joint TTDPM tests should thus investigate potential differences for specific ACL groups.

We reported mean and SD of the TTDPM test results, but for those articles that reported the median and range or quartiles, we have used the method by Wan et al. [17] to estimate mean values. Some studies failed to report relevant data entirely. This did not however affect the overall results of our analyses because those studies with missing data were not eligible for inclusion in the respective meta-analyses.

We did not include studies in the overall evidence synthesis if they received an RoB rating of “inadequate”. The latest COSMIN (2018) guidelines state however that studies with an overall RoB rating of “inadequate” can be included if their results are similar to those of other studies with a higher RoB rating. An additional concern regarding rating of RoB is the questions pertaining to flaws in the study design and statistical methods. Studies could be rated as “inadequate”, “doubtful” or “very good” for these two questions, but not ”adequate”. Many studies thus received a rating of “doubtful” when they may have in fact received a rating of “adequate” had that option been available for those questions. This would have potentially led to the inclusion of more studies in the respective analyses and could have affected our results.

### Future research

Future studies wishing to develop knee joint TTDPM tests should determine the influence of starting angle, movement direction, angular velocity and body position on test outcomes, with full reporting of procedures. Innovative functional assessments of proprioceptive acuity in multiple joints are also encouraged, especially considering clinical implementation. Other relevant information to report includes participant characteristics such as their quantity, age, sex, activity level, type of injury (and surgery), time since injury, side dominance and stability between tests for reliability analyses. When reporting results, the mean and SD (given that the result is normally distributed) should be clearly presented, as well as correlation coefficient when relevant.

## Conclusions

Existing tests of knee joint TTDPM among individuals with ACL injury lack either sufficient quality or a sufficient level of evidence for their reliability, validity and responsiveness. Significantly poorer TTDPM for knees with ACL injury compared to those of asymptomatic controls from a starting angle of 15°, as well as trends towards significance in some of our other meta-analyses, do nevertheless encourage the development of standardised methods. Primary studies investigating the influence of modifiable test components, e.g. starting knee angle, motion direction and angular velocity, on relevant PMPs are therefore recommended to inform evidence-based practice. Standardised, reliable, valid and responsive knee TTDPM tests would facilitate the identification of proprioceptive deficits post-ACL injury and the effectiveness of interventions over time. Until such tests are established, however, outcomes from knee TTDPM tests among individuals with ACL injury should be interpreted with caution.

## Supplementary Information

Below is the link to the electronic supplementary material.

**Additional file 1: Table S1.** Criteria for evaluating the quality of the psychometric properties.

**Additional file 2: Table S2.** Levels of evidence rating for the quality of the psychometric properties.

**Additional file 3: Table S3.** Study characteristics.

**Additional file 4: Table S4.** Risk of bias (RoB) ratings assessed based on the COnsensus-based Standards for the selection of health Measurement INstruments (COSMIN) checklist for each psychometric property (PMP).

**Additional file 5: Table S5.** Convergent validity.

**Additional file 6: Table S6.** Known-groups validity.

**Additional file 7: Table S7.** Discriminative validity.

**Additional file 8: Table S8.** Responsiveness between subgroups.

**Additional file 9: Table S9.** Responsiveness to intervention.

## Data Availability

The datasets used and/or analysed during the current study are available from the corresponding author on reasonable request.

## Abbreviations

ACL: Anterior cruciate ligament
ACLD: Anterior cruciate ligament-deficient
ACLR: Anterior cruciate ligament-reconstructed
AMED: Allied and Complementary Medicine
CENTRAL: The Cochrane Central Register of Controlled Trials
CI: Confidence interval
CINAHL: The Cumulative Index to Nursing and Allied Health Literature
COSMIN: COnsensus-based Standards for the selection of health Measurement Instruments
JPS: Joint position sense
MD: Mean difference
PMPs: Psychometric properties
PRISMA: Preferred Reporting Items for Systematic Reviews and Meta-Analysis
PROSPERO: International Prospective Register of Systematic Reviews
RoB: Risk of bias
SD: Standard deviation
SEM: Standard error of measurement
TTDPM: Threshold to detect passive motion

## References

[1] Needle A, Lepley A, Grooms D (2017). Central nervous system adaptation after ligamentous injury: a summary of theories, evidence, and clinical interpretation. Sports Med.

[2] Proske U, Gandevia SC (2012). The proprioceptive senses: their roles in signaling body shape, body position and movement, and muscle force. Physiol Rev.

[3] Han J, Waddington G, Adams R, Anson J, Liu Y (2016). Assessing proprioception: a critical review of methods. J Sport Health Sci.

[4] Relph N, Herrington L, Tyson S (2014). The effects of ACL injury on knee proprioception: a meta-analysis. Physiotherapy.

[5] Kim HJ, Lee JH, Lee DH (2017). Proprioception in patients with anterior cruciate ligament tears: a meta-analysis comparing injured and uninjured limbs. Am J Sports Med.

[6] Wiggins AJ, Grandhi RK, Schneider DK, Stanfield D, Webster KE, Myer GD (2016). Risk of secondary injury in younger athletes after anterior cruciate ligament reconstruction: a systematic review and meta-analysis. Am J Sports Med.

[7] Poulsen E, Goncalves GH, Bricca A, Roos EM, Thorlund JB, Juhl CB (2019). Knee osteoarthritis risk is increased 4–6 fold after knee injury—a systematic review and meta-analysis. Br J Sports Med.

[8] Arumugam A, Strong A, Tengman E, Röijezon U, Häger CK (2019). Psychometric properties of knee proprioception tests targeting healthy individuals and those with anterior cruciate ligament injury managed with or without reconstruction: a systematic review protocol. BMJ Open.

[9] Moher D, Shamseer L, Clarke M, Ghersi D, Liberati A, Petticrew M (2015). Preferred reporting items for systematic review and meta-analysis protocols (PRISMA-P) 2015 statement. Syst Rev.

[10] Shamseer L, Moher D, Clarke M, Ghersi D, Liberati A, Petticrew M (2015). Preferred reporting items for systematic review and meta-analysis protocols (PRISMA-P) 2015: elaboration and explanation. BMJ.

[11] Strong A, Arumugam A, Tengman E, Röijezon U, Häger CK (2021). Properties of knee joint position sense tests for anterior cruciate ligament injury: a systematic review and meta-analysis. Orthop J Sports Med.

[12] Mokkink LB, de Vet HCW, Prinsen CAC, Patrick DL, Alonso J, Bouter LM (2018). COSMIN Risk of Bias checklist for systematic reviews of Patient-Reported Outcome Measures. Qual Life Res.

[13] Prinsen CAC, Mokkink LB, Bouter LM, Alonso J, Patrick DL, de Vet HCW (2018). COSMIN guideline for systematic reviews of patient-reported outcome measures. Qual Life Res.

[14] Kroman SL, Roos EM, Bennell KL, Hinman RS, Dobson F (2014). Measurement properties of performance-based outcome measures to assess physical function in young and middle-aged people known to be at high risk of hip and/or knee osteoarthritis: a systematic review. Osteoarthritis Cartilage.

[15] Borenstein M, Hedges LV, Higgins JP, Rothstein HR (2010). A basic introduction to fixed-effect and random-effects models for meta-analysis. Res Synth Methods.

[16] Takeshima N, Sozu T, Tajika A, Ogawa Y, Hayasaka Y, Furukawa TA (2014). Which is more generalizable, powerful and interpretable in meta-analyses, mean difference or standardized mean difference?. BMC Med Res Methodol.

[17] Wan X, Wang W, Liu J, Tong T (2014). Estimating the sample mean and standard deviation from the sample size, median, range and/or interquartile range. BMC Med Res Methodol.

[18] Deeks JJ, Higgins JPT, Altman DG, (editors). Chapter 10: Analysing data and undertaking meta-analyses. In: Higgins JPT, Thomas J, Chandler J, Cumpston M, Li T, Page MJ, Welch VA (editors) Cochrane Handbook for Systematic Reviews of Interventions version 60 (updated July 2019) Cochrane, 2019. www.training.cochrane.org/handbook.

[19] Ioannidis JP, Trikalinos TA (2007). The appropriateness of asymmetry tests for publication bias in meta-analyses: a large survey. CMAJ.

[20] Pap G, Machner A, Nebelung W, Awiszus F (1999). Detailed analysis of proprioception in normal and ACL-deficient knees. J Bone Joint Surg Br.

[21] Fridèn T, Roberts D, Zätterström R, Lindstrand A, Moritz U (1999). Proprioceptive defects after an anterior cruciate ligament rupture—the relation to associated anatomical lesions and subjective knee function. Knee Surg Sports Traumatol Arthrosc.

[22] Roberts D, Andersson G, Friden T (2004). Knee joint proprioception in ACL-deficient knees is related to cartilage injury, laxity and age: a retrospective study of 54 patients. Acta Orthop Scand.

[23] Roberts D, Ageberg E, Andersson G, Friden T (2007). Clinical measurements of proprioception, muscle strength and laxity in relation to function in the ACL-injured knee. Knee Surg Sports Traumatol Arthrosc.

[24] Beynnon BD, Ryder SH, Konradsen L, Johnson RJ, Johnson K, Renström PA (1999). The effect of anterior cruciate ligament trauma and bracing on knee proprioception. Am J Sports Med.

[25] Borsa PA, Lephart SM, Irrgang JJ, Safran MR, Fu FH (1997). The effects of joint position and direction of joint motion on proprioceptive sensibility in anterior cruciate ligament-deficient athletes. Am J Sports Med.

[26] Co FH, Skinner HB, Cannon WD (1993). Effect of reconstruction of the anterior cruciate ligament on proprioception of the knee and the heel strike transient. J Orthop Res.

[27] Corrigan JP, Cashman WF, Brady MP (1992). Proprioception in the cruciate deficient knee. J Bone Joint Surg Br.

[28] Courtney CA, Atre P, Foucher KC, Alsouhibani AM (2019). Hypoesthesia after anterior cruciate ligament reconstruction: the relationship between proprioception and vibration perception deficits in individuals greater than one year post-surgery. Knee.

[29] Cronström A (2018). Is poor proprioception associated with worse movement quality of the knee in individuals with anterior cruciate ligament deficiency or reconstruction?. J Phys Ther Sci.

[30] Cronström A, Roos EM, Ageberg E (2017). Association between sensory function and hop performance and self-reported outcomes in patients with anterior cruciate ligament injury. Open Access J Sports Med.

[31] Fischer-Rasmussen T, Jensen PE (2000). Proprioceptive sensitivity and performance in anterior cruciate ligament-deficient knee joints. Scand J Med Sci Sports.

[32] Nishiwaki GA, Tanaka K, Urabe Y (2007). The effect of muscle strength on proprioceptive function after anterior cruciate ligament reconstruction of the knee. Jpn J Phys Fitness Sports Med.

[33] Reider B, Arcand MA, Diehl LH, Mroczek K, Abulencia A, Stroud CC (2003). Proprioception of the knee before and after anterior cruciate ligament reconstruction. Arthroscopy.

[34] Risberg MA, Beynnon BD, Peura GD, Uh BS (1999). Proprioception after anterior cruciate ligament reconstruction with and without bracing. Knee Surg Sports Traumatol Arthrosc.

[35] Barrack RL, Skinner HB, Buckley SL (1989). Proprioception in the anterior cruciate deficient knee. Am J Sports Med.

[36] Cronström A, Ageberg E (2014). Association between sensory function and medio-lateral knee position during functional tasks in patients with anterior cruciate ligament injury. BMC Musculoskelet Disord.

[37] Lee HM, Cheng CK, Liau JJ (2009). Correlation between proprioception, muscle strength, knee laxity, and dynamic standing balance in patients with chronic anterior cruciate ligament deficiency. Knee.

[38] Ozenci AM, Inanmaz E, Ozcanli H, Soyuncu Y, Samanci N, Dagseven T (2007). Proprioceptive comparison of allograft and autograft anterior cruciate ligament reconstructions. Knee Surg Sports Traumatol Arthrosc.

[39] Valeriani M, Restuccia D, Di Lazzaro V, Franceschi F, Fabbriciani C, Tonali P (1996). Central nervous system modifications in patients with lesion of the anterior cruciate ligament of the knee. Brain.

[40] Viggiano D, Corona K, Cerciello S, Vasso M, Schiavone-Panni A (2014). The kinematic control during the backward gait and knee proprioception: insights from lesions of the anterior cruciate ligament. J Hum Kinet.

[41] Fonseca ST, Ocarino JM, Silva PL, Guimarães RB, Oliveira MC, Lage CA (2005). Proprioception in individuals with ACL-deficient knee and good muscular and functional performance. Res Sports Med.

[42] Laboute E, Verhaeghe E, Ucay O, Minden A (2019). Evaluation kinaesthetic proprioceptive deficit after knee anterior cruciate ligament (ACL) reconstruction in athletes. J Exp Orthopaedics.

[43] Arockiaraj J, Korula RJ, Oommen AT, Devasahayam S, Wankhar S, Velkumar S (2013). Proprioceptive changes in the contralateral knee joint following anterior cruciate injury. Bone Joint J.

[44] Fridén T, Roberts D, Zätterström R, Lindstrand A, Moritz U (1997). Proprioception after an acute knee ligament injury: a longitudinal study on 16 consecutive patients. J Orthop Res.

[45] Ma Y, Deie M, Iwaki D, Asaeda M, Fujita N, Adachi N (2014). Balance ability and proprioception after single-bundle, single-bundle augmentation, and double-bundle ACL reconstruction. Sci World J.

[46] Nagai T, Heebner N, Sell T, Nakagawa T, Fu F, Lephart S (2013). Restoration of sagittal and transverse plane proprioception following anatomic double-bundle ACL reconstruction. Knee Surg Sports Traumatol Arthrosc.

[47] Nakamae A, Ochi M, Deie M, Adachi N, Shibuya H, Ohkawa S (2014). Clinical outcomes of second-look arthroscopic evaluation after anterior cruciate ligament augmentation: comparison with single- and double-bundle reconstruction. Bone Joint J.

[48] Pap G, Machner A, Awiszus F (1997). Proprioceptive deficits in anterior cruciate ligament deficient knees: Do they really exist?. Sports Exerc Injury.

[49] Risberg MA, Holm I, Myklebust G, Engebretsen L (2007). Neuromuscular training versus strength training during first 6 months after anterior cruciate ligament reconstruction: a randomized clinical trial. Phys Ther.

[50] Zandiyeh P, Küpper JC, Mohtadi NGH, Goldsmith P, Ronsky JL (2019). Effect of stochastic resonance on proprioception and kinesthesia in anterior cruciate ligament reconstructed patients. J Biomech.

[51] Angoules AG, Mavrogenis AF, Dimitriou R, Karzis K, Drakoulakis E, Michos J (2011). Knee proprioception following ACL reconstruction; a prospective trial comparing hamstrings with bone-patellar tendon-bone autograft. Knee.

[52] Bonfim TR, Grossi DB, Paccola CAJ, Barela JA (2009). Effect of additional sensory information in the proprioception and postural control of individuals with ACL lesion. Acta Ortopédica Brasileira.

[53] Bonfim TR, Jansen Paccola CA, Barela JA (2003). Proprioceptive and behavior impairments in individuals with anterior cruciate ligament reconstructed knees. Arch Phys Med Rehabil.

[54] Fridén T, Roberts D, Zätterström R, Lindstrand A, Moritz U (1996). Proprioception in the nearly extended knee. Measurements of position and movement in healthy individuals and in symptomatic anterior cruciate ligament injured patients. Knee Surg Sports Traumatol Arthrosc.

[55] Jensen TØ, Fischer-Rasmussen T, Kjær M, Magnusson SP (2002). Proprioception in poor- and well-functioning anterior cruciate ligament deficient patients. J Rehabil Med.

[56] Lee BI, Kwon SW, Kim JB, Choi HS, Min KD (2008). Comparison of clinical results according to amount of preserved remnant in arthroscopic anterior cruciate ligament reconstruction using quadrupled hamstring graft. Arthroscopy.

[57] Lephart SM, Kocher MS, Fu FH, Borsa PA, Harner CD (1992). Proprioception following anterior cruciate ligament reconstruction. J Sport Rehab.

[58] MacDonald PB, Hedden D, Pacin O, Sutherland K (1996). Proprioception in anterior cruciate ligament-deficient and reconstructed knees. Am J Sports Med.

[59] Roberts D, Fridén T, Stomberg A, Lindstrand A, Moritz U (2000). Bilateral proprioceptive defects in patients with a unilateral anterior cruciate ligament reconstruction: a comparison between patients and healthy individuals. J Orthop Res.

[60] Roberts D, Fridén T, Zätterström R, Lindstrand A, Moritz U (1999). Proprioception in people with anterior cruciate ligament-deficient knees: comparison of symptomatic and asymptomatic patients. J Orthop Sports Phys Ther.

[61] Shidahara H, Deie M, Niimoto T, Shimada N, Toriyama M, Adachi N (2011). Prospective study of kinesthesia after ACL reconstruction. Int J Sports Med.

[62] Courtney C, Rine RM, Kroll P (2005). Central somatosensory changes and altered muscle synergies in subjects with anterior cruciate ligament deficiency. Gait Posture.

[63] Fischer-Rasmussen T, Jensen TØ, Kjær M, Krogsgaard M, Dyhre-Poulsen P, Magnusson SP (2001). Is proprioception altered during loaded knee extension shortly after ACL rupture?. Int J Sports Med.

[64] Gupta RK, Aggarwal S, Aggarwal V, Garg SK, Kumar S (2010). Preserved insertions of the semitendinosus and gracilis tendons (STG) in ACL reconstruction: a new surgical technique with preliminary results. Curr Orthop Pract.

[65] Shen M, Che S, Ye D, Li Y, Lin F, Zhang Y (2019). Effects of backward walking on knee proprioception after ACL reconstruction. Physiother Theory Pract.

[66] Ageberg E, Björkman A, Rosén B, Roos EM (2012). Principles of brain plasticity in improving sensorimotor function of the knee and leg in patients with anterior cruciate ligament injury: a double-blind randomized exploratory trial. BMC Musculoskelet Disord.

[67] Valeriani M, Restuccia D, Di Lazzaro V, Franceschi F, Fabbriciani C, Tonali P (1999). Clinical and neurophysiological abnormalities before and after reconstruction of the anterior cruciate ligament of the knee. Acta Neurol Scand.

[68] Beynnon BD, Johnson RJ, Naud S, Fleming BC, Abate JA, Brattbakk B (2011). Accelerated versus nonaccelerated rehabilitation after anterior cruciate ligament reconstruction: a prospective, randomized, double-blind investigation evaluating knee joint laxity using roentgen stereophotogrammetric analysis. Am J Sports Med.

[69] Proske U, Gandevia SC (2018). Kinesthetic Senses. Compr Physiol.

[70] Nakamae A, Adachi N, Ishikawa M, Nakasa T, Ochi M (2017). No evidence of impaired proprioceptive function in subjects with anterior cruciate ligament reconstruction: a systematic review. J ISAKOS.

