# Supplementary material for: Properties of tests for knee joint threshold to detect passive motion following anterior cruciate ligament injury: a systematic review and meta-analysis
Source: J Orthop Surg Res. 2022 Mar 4;17:134. doi: 10.1186/s13018-022-03033-4 (PMC8895768; doi:10.1186/s13018-022-03033-4)
Supplement: Supplementary file 1 — Additional file 1: Table S1. Criteria for evaluating the quality of the psychometric properties. [file 13018_2022_3033_MOESM1_ESM.docx]

**SUPPLEMENTAL TABLE S1**

Criteria for evaluating the quality of the psychometric properties

| Measurement property | Rating^a^ | Criteria |
| --- | --- | --- |
| Reliability | + | Intraclass correlation coefficient or weighted Kappa > 0.70 |
|  | ? | Intraclass correlation coefficient or weighted Kappa not reported |
|  | - | Intraclass correlation coefficient or weighted Kappa < 0.70 |
| Measurement error | + | Smallest detectable change or limits of agreement < minimal important change^b^ |
|  | ? | Minimal important change not defined |
|  | - | Smallest detectable change or limits of agreement > minimal important change^b^ |
| Hypothesis testing for construct validity | +  ? | The results are in accordance with the hypothesis^c^  Hypothesis is not defined (by the review committee) |
|  | - | The result is not in agreement with the hypothesis^c^ |
| Criterion validity | + | Correlation with gold/reference standard > 0.70 or area under the curve > 0.70 |
|  | ? | Not all information for ‘+’ reported |
|  | - | Correlation with gold/reference standard < 0.70 or area under the curve < 0.70 |
| Responsiveness | + | The results are in agreement with the hypothesis^c^ or area under the curve > 0.70 |
|  | ? | Hypothesis is not defined (by the review committee) |
|  | - | The results are not in agreement with the hypothesis^c^ or area under the curve < 0.70 |
| The criteria list has been adapted from Prinsen et al.^81^  ^a^Rating: ‘+’ = sufficient, ‘?’ = indeterminate, ‘-’ = sufficient.  ^b^This rating of evidence may be obtained from different studies.  ^c^The findings of all studies must be compiled together and then it must be decided if 75% of the findings are in agreement with the hypotheses or not. | | |
