# Supplementary material for: Properties of tests for knee joint threshold to detect passive motion following anterior cruciate ligament injury: a systematic review and meta-analysis
Source: J Orthop Surg Res. 2022 Mar 4;17:134. doi: 10.1186/s13018-022-03033-4 (PMC8895768; doi:10.1186/s13018-022-03033-4)
Supplement: Supplementary file 2 — Additional file 2: Table S2. Levels of evidence rating for the quality of the psychometric properties. [file 13018_2022_3033_MOESM2_ESM.docx]

**SUPPLEMENTAL TABLE S2**

Levels of evidence rating for the quality of the psychometric properties

| Level | Rating^a^ | Criteria^b^ |
| --- | --- | --- |
| Strong | +++ or - - - | Test^c^ was evaluated in multiple studies of adequate risk of bias rating or one study of very good risk of bias rating (implying a low risk of bias) |
| Moderate | ++ or - - | Test was evaluated in multiple studies of doubtful risk of bias rating or one study of adequate risk of bias rating |
| Limited | + or - - - | Test was evaluated in one study of doubtful risk of bias rating |
| Conflicting | ± | Test was evaluated in studies with contradictory findings |
| Unknown | ? | Test was evaluated in studies of inadequate risk of bias rating or not investigated at all |
| Adapted from Kroman et al.^60^  ^a^Ratings: ‘+’ = sufficient, ‘?’ = indeterminate, ‘-’ = insufficient.  ^b^Modified using the 4-point scoring system of the updated COSMIN checklist.^70^  ^c^A specific test of knee joint position sense. | | |
