# Supplementary material for: Properties of tests for knee joint threshold to detect passive motion following anterior cruciate ligament injury: a systematic review and meta-analysis
Source: J Orthop Surg Res. 2022 Mar 4;17:134. doi: 10.1186/s13018-022-03033-4 (PMC8895768; doi:10.1186/s13018-022-03033-4)
Supplement: Supplementary file 3 — Additional file 3: Table S3. Study characteristics. [file 13018_2022_3033_MOESM3_ESM.docx]

**SUPPLEMENTAL TABLE S3**

Study characteristics

| **Reference** | **Study design** | **Population** | |  | **TTDPM methods** | | | | | | | |
| --- | --- | --- | --- | --- | --- | --- | --- | --- | --- | --- | --- | --- |
|  |  | **ACLD/ACLR/CTRL** | **N (female)** | **Age, years**  **Mean ± SD/**  **Mdn (range)** | **Time since injury/surgery** | **Other injuries** | **Angular velocity (°/s)** | **Movement direction (flex/ext)** | **Body position** | **Equipment** | **No. of trials** | **SA (°)** |
| Ageberg et al. (2012) | Exploratory randomized clinical | ACLR  ACLD | 24 (NR)^a^  15 (NR)^a^ | 25 (5.6) | ≥ 16 weeks  ≥ 10 weeks | Yes | NR | Flex/ext | Side lying | Motor pulley | 3/direction | 20 |
| Angoules et al. (2011) | Prospective | ACLR  ACLR | 20 (4)  20 (2) | 31 (17-54) | 6 (2-24) months | No | 2 | Flex/ext | Sitting | Isokinetic dynamometer | 6/each leg | 15/45 |
| Arockiaraj et al. (2013) | Cross-  sectional | ACLD  CTRL | 25 (NR) 25 (NR) | 28 (18-38)  28.3 (21-33) | 29 (2-108) months | No | 0.2 | Ext | Sitting | Electronic goniometer | 3/direction | 30/70 |
| Barrack et al. (1989) | Cross- sectional | ACLD  CTRL | 12 (2)  10 (NR) | 25 (21-30)  Age-matched | NR | No | 0.5 | Ext | Sitting | Motor pulley | 5/each leg | 40 |
| Beynnon et al. (1999) | Cross-  sectional | ACLD | 20 (7) | 40 (26-53) | 5.5 (1.2-21.8)  years | Yes | 0.1 | Flex/ext | Sitting | Motor pulley | 6/each leg | 45 |
| Beynnon et al. (2011) | Randomized controlled trial | ACLR  ACLR | 19  17 | 29.7 ± 10.1  30.2 ± 9.9 | 24 months | Yes | 0.1 | Flex/ext | Sitting | Motor pulley | 6/each leg | NR |
| Bonfim et al. (2009) | Cross-  sectional | ACLD  CTRL | 28 (NR)  28 (NR) | 23 ± 4  22 ± 2 | 20 (±10) months | Yes | 0.5 | Flex/ext | Supine | CPM system | 3/direction | 15/45 |
| Bonfim et al. (2003) | Cross- sectional | ACLR  CTRL | 10 (3)  10 (3) | 24.4 ± 4.5  24.4 ± 3.0 | 18 (12-30) months | No | 0.5 | Flex/ext | Supine | CPM system | 3/direction | 0/15/30/45/60 |
| Borsa et al. (1997) | Cross-  sectional | ACLD | 29 (14) | 28.7 ± 1.7 | 41.7 ± 11.7 (2-228) months | Yes | 0.5 | Flex/ext | Sitting | Motor driven rotational transducer | NR | 15/45 |
| Co et al. (1993) | Cross- sectional | ACLR  CTRL | 10 (5)  10 (5) | 27 (9-37)  25 (21-29) | 31.6 months | Yes | 0.5 | Flex | Sitting | Motor pulley | 5/each leg | 40 |
| Corrigan et al. (1992) | Cross- sectional | ACLD  CTRL | 20 (NR)  17 (0) | 30 (22-40)  28 (20-32) | 5.25 (2-14) months | No | 0.3 | NR | Sitting | Motor pulley | 5/each leg | 35 |
| Courtney et al. (2005) | Cross-  sectional | ACLD  ACLD  ACLDCTRL | 3 (NR)  4 (NR)  10 (NR)  7 (6) | 34.5 ± 8.6  27 ± 4.1 | 2-214 months | Yes | 0.5 | Flex/ext | Sitting | Motor pulley | 1/each leg | 40 |
| Courtney et al. (2019) | Cross-  sectional | ACLR  CTRL | 20 (10)  6 (NR) | 27 ± 6 (20-47)  27 ± 5.5 | NR | No | 0.5 | Flex/ext | Sitting | Motor pulley | 3/each leg | 45 |
| Cronström et al. (2014) | Cross-  sectional | ACLD/  ACLR | 51 (23) | F: 23 (4.0)  M: 26 (6.1) | F: 45 (42.1) weeks  M: 66 (57.9) | Yes | 0.5 | Flex/ext | Side lying | Biothesiometer | 3/direction | 20 |
| Cronström et al. (2017) | Cross- sectional/ exploratory | ACLR  ACLD  CTRL | 33 (13)  20 (9) | 24 (18–35)  23 (18–35) | 19.3 (17.9–35.5)  20.2 (11.4–41.5) | NR | 0.5 | Flex/ext | Side lying | Motor pulley | 3/direction | 20 |
| Cronström (2018) | Cross-  sectional | ACLD  ACLR | 13 (4%)  38 (19%) | 27 ± 5.7  23 ± 5.2 | 21.29 (16.6–52.6)  27.8 (19.7–47.1)  (weeks) | Yes | 1 | Flex/ext | Side lying | Electrical motor | 3 | 20 |
| Fischer-Rasmussen et al. (2000) | Cross- sectional | ACLD  ACLR  CTRL | 20 (9)  18 (9)  20 (9) | 27 ± 5  27 ± 4 | NR | NR | 0.5 | Flex | Supine | Customized device | NR | 20 |
| Fischer-Rasmussen et al. (2001) | Cross-  sectional | ACLD  CTRL | 10 (4)  15 (11) | 27.3 ± 6.1  27.7 ± 6.7 | 7.5 ± 2.8 weeks | Yes | 0.5 | Flex | Supine | Electronic  goniometer | NR | 20 |
| Fonseca et al. (2005) | Cross- sectional | ACLD  CTRL | 11 (2)  11 (NR) | 26.45 (7.78)  27.35 (7.65) | 7.73 (3.17) months | No | 2 | Ext | Sitting | Isokinetic dynamometer | 3 | 35 |
| Fridén et al. (1996) | Cross- sectional | ACLD  CTRL | 20 (6)  19 (5) | 26 (18-39)  25 (20-37) | 3.6 (1-16) years | Yes | 0.5 | Flex/ext | Side lying | Motor pulley | 3 | 20/40 |
| Fridén et al. (1997) | Quasi-  experimental | ACLD  CTRL | 16 (5)  19 (5) | 26 (15-36)  25 (20-37) | 1-12 months | No | 0.5 | Flex/ext | Side lying | Motor pulley | 3/each leg | 20/40 |
| Fridén et al. (1999) | Prospective and longitudinal | ACLD | 16 (5) | 26 (15-36) | NR | Yes | 0.5 | Flex/ext | Side lying | Motor pulley | 3/each leg | 20/40 |
| Gupta et al. (2010) | Retrospective | ACLR | 45 (9) | 25.4 (18-44) | 40.6 (34-50) months | NR | NR | NR | NR | NR | NR | NR |
| Jensen et al. (2002) | Cross- sectional | ACLD  ACLD | 7 (1)  7 (4) | 31.1 ± 4.5  30.1 ± 3.6 | 4.4 ± 2.8 years  6.4 ± 2.1 years | Yes | 0.5 | Ext | Supine | Customized device | 5/each leg  2 test trials | 20 |
| Laboute et al. (2019) | Cross- sectional | ACLR  ACLR  CTRL | 32 (7)  32 (10)  32 (10) | 24.9 ± 5.9  24.9 ± 5.3  26.2 ± 6.2 | 21-35 days  3-9 months | No | 4 | Flex | Sitting | Isokinetic dynamometer | 10/each leg | 15 |
| Lee et al. (2008) | Cross-  sectional | ACLR | 16 (NR) | 35.1 (24-57) | 35.1 months | Yes | 0.5 | Ext | NR | CPM and goniometer | 5/each leg | 15/30/45 |
| Lee et al. (2009) | Cross-  sectional | ACLD | 12 (2) | 23.1 (20-26) | 12.8 (9-24) months | No | 0.5 | Flex/ext | Sitting | Customized  device | 3 | 45 |
| Lephart et al. (1992) | Cross- sectional | ACLR | 12 (8) | 23.2 ± 7.0 | 11-26 months | No | 0.5 | Flex/ext | Sitting | Customized device | NR | 15/45 |
| Ma et al. (2014) | Retrospective | ACLR  ACLR  ACLR | 20 (10)  21 (10)  26 (15) | 25.2 ± 1.3  29.5 ± 1.3  26.7 ± 1.4 | NR  NR  NR | No | 0.2 | Flex/ext | Sitting | Customized  device | NR | 45 |
| MacDonald et al. (1996) | Cross- sectional | ACLD  ACLR  ACLR  CTRL | 10 (4)  8 (3)  8 (3)  6 (NR) | 28.5 (15-41)  25.8 (18-32)  26.4 (21-39)  30 (23-39) | 66.8 (5-193)  24 (17-30)  31 (18-50)  months | No | 0.5 | Flex/ext | Sitting | Electrical motor | 5/each leg  2 test trials | 30/40 |
| Nagai et al. (2013) | Cross- sectional | ACLR  CTRL | 11 (4)  11 (4) | 23.1 ± 4.8  22.5 ± 3.2 | 12.5–15 months | No | 0.25 | Flex/ext | Sitting | Electrical motor | 5/direction | 15 |
| Nakamae et al. (2014) | Prospective | ACLR  ACLR  ACLR | 61 (34)  82 (35)  73 (44) | 24.6 (11-58)  24.8 (13-55)  26.6 (12-56) | NR  NR  NR | No | 0.2 | Flex/ext | Sitting | Customized  device | 4/direction | 15/45 |
| Nishiwaki et al. (2007) | Prospective cohort | ACLR | 16 (10) | 28 ± 2.3 | NR | No | 0.5 | Flex/ext | Sitting | Electrical motor | NR | 15/45 |
| Ozenci et al. (2007) | Retrospective  clinical | ACLR  ACLR  ACLD  CTRL | 20 (0)  20 (4)  20 (2)  20 (3) | 29.5 ± 6.9  30.2 ± 4.6  29.0 ± 5.4  27.6 ± 2.6 | 16.5 ± 5.5  25.6 ± 13.0  12.5 ± 3.6 | Unclear | 1 | Flex/ext | Sitting | Electrical motor | 10 | 15 |
| Pap et al. (1997) | Cross- sectional | ACLD  CTRL | 20 (6)  20 (9) | 24.5 ± 5.2  25.3 ± 4.8 | 14.2 (9-23) weeks | NR | 0.15m/s | Flex/ext | Sitting | Electrical motor | 10 | 45 |
| Pap et al. (1999) | Cross- sectional | ACLD  CTRL | 20 (6)  15 (6) | 24.5 ± 5.2  25.3 ± 4.8 | 10 (8-14) weeks | No | 0.1-0.85 | Flex/ext | Sitting | Electrical motor | 6/direction/velocities | 45 |
| Reider et al. (2003) | Prospective  cohort | ACLR  CTRL | 26 (15)  26 (13) | 25 (16-48)  25 (18-40) | 8 (2-156) weeks | Yes | 3 | Flex/ext | Sitting | Electric  goniometer | 10/each leg | 15 |
| Risberg et al. (1999) | Prospective  cohort | ACLR  CTRL | 20 (12)  10 (5) | 35 (22-47)  33 (22-41) | 4.6 months (to op) and follow-up 24 months | Yes | 0.5 | Flex/ext | Sitting | Motor pulley | 12 trials | 15 |
| Risberg et al. (2007) | Randomized clinical trial | ACLR  ACLR | 39 (13)  35 (14) | F:27.2 (20.6-37.9)  M:27.7 (16.7-39.6)  F: 26.5 (19.8-38)  M:31.2 (19.4-40.3) | Max. 3 years | Yes | 0.5 | Flex/ext | NR | Customized device | 3/direction | 15 |
| Roberts et al. (1999) | Nonrandomized  prospective | ACLD  ACLD  CTRL | 17 (7)  20 (6)  19 (5) | 28.8 ± 5.6  26.6 ± 6.1  25.6 ± 3.7 | 6.5 ± 1.3 years  3.6 ± 3.7 years | NR | 0.5/10 | Flex/ext | Side lying | Motor pulley | 3/direction | 20/40 |
| Roberts et al. (2000) | Cross-  sectional | ACLR  CTRL | 20 (5)  19 (5) | 27 (19-38)  25 (20-37) | 2 years | No | 0.5 | Flex/ext | Side lying | Motor pulley | 3/angle/direction | 20/40 |
| Roberts et al. (2004) | Retrospective | ACLD | 54 (20) | 28 (16-42) | 2.7 (2.7) years | Yes | 0.5 | Flex/ext | Side lying | Customized device | 4/each position | 20/40 |
| Roberts et al. (2004) | Intervention | ACLD  CTRL | 36 (18)  24 (13) | 25.9 (16–35)  23.7 (20–32) | NR | Yes | 0.5 | Flex/ext | Side lying | Customized device | 3 trials | 30/40 |
| Roberts et al. (2007) | Cross-  sectional | ACLD | 36 (18) | 25.9 ± 5.4 | NR | Yes | 0.5 | Flex/ext | Side lying | Customized  device | 3 trials | 30/40 |
| Shen et al. (2019) | Intervention | ACLR  ACLR  ACLR  ACLR  ACLR | 10 (5)  11 (5)  11 (5)  10 (4)  10 (3) | 36.6 ± 12.1  37.5 ± 9.39  34 ± 10.29  32.9 ± 11.45  35.5 ± 10.1 | 3-6 months | No | 0-1 | Flex/ext | Supine | Customized  device | 3/direction | 20/50/80 |
| Shidahara et al. (2011) | Prospective | ACLR | 31 (18) | 22.2 ± 9.7 | Unclear | Unclear | 0.1/0.2 | Flex/ext | Sitting | Motor pulley | 8/position/direction | 15/45 |
| Valeriani et al. (1996) | Cross- sectional | ACLD | 19 (NR) | 28 ± 4.09 | 1-8 years | No | NR | Ext | Sitting | Motor pulley | 5/each leg | 40 |
| Valeriani et al. (1999) | Quasi-  experimental | ACLR | 7 (NR) | NR | NR | No | NR | Ext | Sitting | Motor pulley | 5/each leg | 40 |
| Viggiano et al. (2014) | Quasi-  experimental | ACLD  ACLR  CTRL | 15 (0)  15 (0)  15 (0) | 30 ± 4.8  24 ± 4.6  25 ± 3.8 | Beyond 9 months | No | 0.5 | Flex/ext | Sitting | Motor pulley | Min. 3/direction | 90 |
| Zandiyeh et al. (2019) | Cross- over | ACLR  CTRL | 19 (19)  28 (28) | 24 ± 5  25 ± 4 | 3 months | No | 0.25 | Flex/ext | Sitting | Isokinetic dynamometer | 5/each leg | 15 |
| ^a^49% females across populations but unclear for each group.  N.B. Quasi-experimental study design contains a number of study designs, including pretest-posttest and combinations of test designs within the same study  Abbreviations: ACLD = anterior cruciate ligament-deficient; ACLR = anterior cruciate ligament-reconstructed; CPM device = Continuous Passive Motion device; CTRL = control; Ext = extension; Flex = flexion; IQR = interquartile range; Mdn = median; NA = not applicable; NR = not reported; SA = starting angle; TTDPM = Threshold to detect passive motion | | | | | | | | | | | | |
