# Supplementary material for: Properties of tests for knee joint threshold to detect passive motion following anterior cruciate ligament injury: a systematic review and meta-analysis
Source: J Orthop Surg Res. 2022 Mar 4;17:134. doi: 10.1186/s13018-022-03033-4 (PMC8895768; doi:10.1186/s13018-022-03033-4)
Supplement: Supplementary file 4 — Additional file 4: Table S4. Risk of bias (RoB) ratings assessed based on the COnsensus-based Standards for the selection of health Measurement INstruments (COSMIN) checklist for each psychometric property (PMP). [file 13018_2022_3033_MOESM4_ESM.docx]

**SUPPLEMENTAL TABLE S4**

Risk of bias (RoB) ratings assessed based on the the COnsensus-based Standards for the selection of health Measurement INstruments (COSMIN) checklist for each psychometric property (PMP).

|  | | | | | | |
| --- | --- | --- | --- | --- | --- | --- |
| Measurement error | | | | | | |
|  | Were patients stable in the interim period on the construct to be measured? | Was the time interval appropriate? | Were the test conditions similar for the measurements? e.g. type of administration, environment, instructions | For continuous scores: Was the SEM, SDC or LoA calculated? | Were there any other important flaws in the design or statistical methods of the study? | Overall RoB |
| Roberts et al. (2004) | 1 | 1 | 2 | 4 | NA | 1 |
| Convergent validity | | | | | | |
|  | Is it clear what the comparator instrument(s) measure(s)? | Were the measurement properties of the comparator instrument(s) sufficient? | Was the statistical method appropriate for the hypotheses to be tested? | Were there any other important flaws in the design or statistical methods of the study? | Overall  RoB |  |
| Barrack (1989) | 4 | 4 | 3 | 1 | 1 |  |
| Beynnon (1999) | 4 | 3 | 3 | 2 | 2 |  |
| Borsa (1997) | 4 | 4 | 3 | 2 | 2 |  |
| Co (1993) | 4 | 4 | 4 | 2 | 2 |  |
| Corrigan (1992) | 4 | 4 | 2 | 2 | 2 |  |
| Courtney (2019) | 4 | 3 | 3 | 2 | 2 |  |
| Cronström (2014) | 4 | 4 | 3 | 1 | 1 |  |
| Cronström (2017) | 4 | 4 | 2 | 2 | 2 |  |
| Cronström (2018)  Fischer-Rasmussen (2000) | 4  4 | 4  3 | 4  2 | 2  2 | 2  2 |  |
| Fridén (1999) | 4  4  4  4  4  4  4  4 | 4  4  4  4  4  4  4  1 | 4  4  4  4  4  4  4  4 | 2  2  2  2  2  2  2  2 | 2 (activity before injury)  2 (Lachman pos/neg)  2 (age)  2 (pivot shift pos/neg)  2 (lesions if med coll lig (yes/no) or meniscus medial, lateral, both (yes/no)  2 (cartilage yes/no)  2 (gender)  1 (subjective rating 1-10) |  |
| Lee (2009) | 4 | 1 | 4 | 2 | 1 |  |
| Nishiwaki (2007) | 4 | 4 | 2 | 2 | 2 |  |
| Ozenci (2007) | 4 | 4 | 1 | 2 | 1 |  |
| Reider (2003) | 4 | 4 | 2 | 2 | 2 |  |
| Risberg (1999) | 4 | 4 | 3 | 2 | 2 |  |
| Roberts (2004) | 4  4  4  4  4  4  4  4  4  4  4 | 4  4  4  1  4  4  4  4  4  4  4 | 3  3  3  3  3  3  3  3  3  3  3 | 2  2  2  2  2  2  2  2  2  2  2 | 2 (activity level before)  2 (activity level after)  2 (age)  1 (subjective function)  2 (Lachman)  2 (lateral cartilage injury)  2 (lateral meniscus injury)  2 (medial cartilage injury)  2 (medial meniscus injury)  2 (medial collateral lig. injury)  2 (gender) |  |
| Roberts (2007) | 4  4 | 4  1 | 4  4 | 2  2 | 2 (single leg hop distance and subjective function)  1 (subjective function) |  |
| Valeriani (1996) | 4 | 1 | 1 | 1 | 1 |  |
| Viggiano (2004) | 4 | 1 | 3 | 1 | 1 |  |
| Known-groups validity | | | | | | |
|  | Was an adequate description provided of important characteristics of the subgroups? | Was the statistical method appropriate for the hypotheses to be tested? | Were there any other important flaws in the design or statistical methods of the study? | Overall  RoB |  |  |
| Angoules (2011) | 2 | 2 | 1 | 1 |  |  |
| Arockiaraj (2013) | 2 | 3 | 2 | 2 |  |  |
| Bonfim (2003) | 3 | 3 | 1 | 1 |  |  |
| Bonfim (2009) | 3 | 3 | 1 | 1 |  |  |
| Co (1993) | 3 | 3 | 4 | 3 |  |  |
| Courtney (2005) | 2 | 2 | 1 | 1 |  |  |
| Courtney (2019) | 4 | 3 | 4 | 3 |  |  |
| Cronström (2018) | 4 | 2 | 1 | 1 |  |  |
| Cronström (2014) | 3 | 2 | 1 | 1 |  |  |
| Fischer-Rasmussen (2000) | 3 | 4 | 2 | 2 |  |  |
| Fonseca (2005) | 4 | 4 | 4 | 4 |  |  |
| Fridén (1996) | 3 | 2 | 1 | 1 |  |  |
| Fridén (1997) | 3 | 2 | 4 | 2 |  |  |
| Jensen (2002) | 4 | 3 | 1 | 1 |  |  |
| Laboute (2019) | 4 | 4 | 4 | 4 |  |  |
| Lee (2008) | 3 | 3 | 1 | 1 |  |  |
| Lephart (1992) | 2 | 3 | 1 | 1 |  |  |
| Ma (2014) | 3 | 4 | 2 | 2 |  |  |
| MacDonald (1996) | 4 | 2 | 1 | 1 |  |  |
| Nagai (2013) | 3 | 2 | 2 | 2 |  |  |
| Nakamae (2014) | 2 | 4 | 2 | 2 |  |  |
| Ozenci (2007) | 4 | 3 | 2 | 2 |  |  |
| Pap (1997) | 2 | 2 | 2 | 2 |  |  |
| Pap (1999) | 2 | 3 | 1 | 1 |  |  |
| Reider (2003) | 3 | 3 | 2 | 2 |  |  |
| Risberg (1999)  Risberg (2007) | 4  4 | 2  3 | 4  2 | 2  2 |  |  |
| Roberts (1999) | 3 | 2 | 1 | 1 |  |  |
| Roberts (2000) | 3 | 2 | 1 | 1 |  |  |
| Viggiano (2014) | 4 | 4 | 1 | 1 |  |  |
| Zandiyeh (2019) | 3 | 4 | 2 | 2 |  |  |
| Discriminative validity | | | | | | |
|  | Was an adequate description provided of important characteristics of the subgroups? | Was the statistical method appropriate for the hypotheses to be tested? | Were there any other important flaws in the design or statistical methods of the study? | Overall  RoB |  |  |
| Angoules (2011) | 2 | 2 | 1 | 1 |  |  |
| Arockiaraj (2013) | 2 | 1 | 2 | 1 |  |  |
| Barrack (1989) | 2 | 4 | 1 | 1 |  |  |
| Beynnon (1999) | 4 | 4 | 2 | 2 |  |  |
| Borsa (1997)  Co (1993)  Courtney (2005)  Courtney (2019) | 3  3  2  4 | 1  3  2  3 | 2  4  1  4 | 1  3  1  3 |  |  |
| Fischer-  Rasmussen (2000)  Fischer-  Rasmussen (2001) | 4  4 | 3  4 | 2  1 | 2  1 |  |  |
| Fonseca (2005) | 4 | 4 | 4 | 4 |  |  |
| Fridén (1996) | 3 | 2 | 1 | 1 |  |  |
| Fridén (1997) | 3 | 2 | 2 | 2 |  |  |
| Gupta (2010) | 2 | 3 | 1 | 1 |  |  |
| Jensen (2002) | 4 | 3 | 1 | 1 |  |  |
| Laboute (2019) | 4 | 4 | 4 | 4 |  |  |
| Lee (2009) | 4 | 3 | 2 | 2 |  |  |
| Lee (2008)  Lephart (1992)  Macdonald (1996)  Nagai (2013)  Nishiwaki (2007)  Ozenci (2007)  Pap (1997)  Pap (1999)  Reider (2003) | 3  2  4  3  3  4  2  2  3 | 1  3  3  2  3  3  2  3  3 | 1  1  1  2  2  2  2  2  2 | 1  1  1  2  2  2  2  2  2 |  |  |
| Risberg (1999)  Roberts (2000)  Shidahara (2011)  Valeriani (1996) | 2  3  4  3 | 1  2  4  1 | 2  2  2  1 | 1  2  2  1 |  |  |
|  | | | | | | |
| Responsiveness between subgroups | | | | | | |
|  | Was an adequate description provided of important characteristics of the subgroups? | Was the statistical method appropriate for the hypotheses to be tested? | Were there any other important flaws in the design or statistical methods of the study? | Overall  RoB |  |  |
| Ageberg (2012) | 4 | 4 | 1 | 1 |  |  |
| Angoules (2011) | 2 | 2 | 1 | 1 |  |  |
| Bonfim (2009) | 3 | 4 | 1 | 1 |  |  |
| Ma (2014) | 3 | 1 | 2 | 1 |  |  |
| Risberg (2007) | 4 | 4 | 2 | 2 |  |  |
| Shen (2019) | 3 | 4 | 2 | 2 |  |  |
| Zandiyeh (2019) | 3 | 4 | 2 | 2 |  |  |
| Responsiveness to intervention | | | | | | |
|  | Was an adequate description provided of the intervention given? | Was the statistical method appropriate for the hypotheses to be tested? | Were there any other important flaws in the design or statistical methods of the study? | Overall  RoB |  |  |
| Beynnon (1999) | 2 | 4 | 2 | 2 |  |  |
| Gupta (2010) | 4 | 3 | 1 | 1 |  |  |
| Lephart (1992) | 4 | 3 | 1 | 1 |  |  |
| Risberg (1999) | 4 | 3 | 4 | 3 |  |  |
| Roberts (2004) | 4 | 1 | 2 | 1 |  |  |
| Shen (2019) | 2 | 2 | 1 | 1 |  |  |
| Shidahara (2011) | 4 | 4 | 2 | 2 |  |  |
| Valeriani (1999) | 2 | 4 | 1 | 1 |  |  |
| Zandiyeh (2019) | 2 | 4 | 2 | 2 |  |  |
| Abbreviations: SEM = Standard Error of Measurement; SDC = Smallest Detectable Change; LoA = Limits of Agreement; ICC = Intraclass Correlation  Coefficient; RoB = Risk of Bias.  Ratings: 1 = inadequate, 2 = doubtful, 3 = adequate, 4 = very good. | | | | | | |
