# Supplementary material for: Properties of tests for knee joint threshold to detect passive motion following anterior cruciate ligament injury: a systematic review and meta-analysis
Source: J Orthop Surg Res. 2022 Mar 4;17:134. doi: 10.1186/s13018-022-03033-4 (PMC8895768; doi:10.1186/s13018-022-03033-4)
Supplement: Supplementary file 5 — Additional file 5: Table S5. Convergent validity. [file 13018_2022_3033_MOESM5_ESM.docx]

**SUPPLEMENTAL TABLE S5**

Convergent validity

|  | **TTDPM test details** | | | |  | **Convergent validity** | |  | **Quality** | |  |
| --- | --- | --- | --- | --- | --- | --- | --- | --- | --- | --- | --- |
|  | **Position** | **Angular velocity** | **Direction** | **SA (°)** |  | **Outcome measure/s** | **r** |  | **PMP** | **Meth.** |  |
|  | Sitting | 0.5°/s | Ext | 40 |  | a) Knee laxity | 0.465 |  | NA | Inadequate |  |
|  | Sitting | 0.1°/s | Flex/ext | 45 |  | a) Knee laxity KT-1000  b) Pivot shift grade | 0.022  0.048 |  | -  - | Doubtful |  |
|  | Sitting | 0.5°/s | Flex  Ext  Flex  Ext  Flex  Ext  Flex  Ext  Flex  Ext | 15  45  15  45  15  45  15  45  15  45  15  45  15  45  15  45  15 45  15  45 |  | a) Hop test  b) TTDPM 15°F  c) TTDPM 45°F  d) TTDPM 15°E  e) TTDPM 45°E | -0.365  -0.365  -0.462  -0.558  -  0.791  0.591  0.593  0.791  -  0.348  0.478  0.591  0.348  -  0.685  0.593  0.478  0.685  - |  | -  -  -  -  +  -  -  +  -  -  -  -  -  -  -  - | Doubtful |  |
|  | Sitting | 0.5°/s | Flex | 40 |  | a) Reproduction of passive motion  b) Relative reproduction  c) Heel strike transient  d) Isokinetic extension | NS  NS NS  NS |  | -  -  -  - | Doubtful |  |
|  | NR | 0.3°/s | NR | 35 |  | a) Threshold for movement detection plotted against hamstring/quadriceps ratio in 11 ACLD patients  b) Position error plotted against hamstring/quadriceps ratio in 11 ACLD patients | -0.74  -0.77 |  | +  + | Doubtful |  |
|  | Sitting | 0.5°/s | Flex/ext | 45 |  | a) VPT lateral femoral condyle  b) VPT lateral malleolus  c) Isometric strength | 0.462  -0.380 |  | ?  -  - | Doubtful |  |
|  | Side lying | 0.5°/s | Flex/ext | 20 |  | Sensory test  a) Single limb mini-squat  b) Stair descending  c) Forward lunge  d) Drop jump  a) Single limb mini-squat  b) Stair descending  c) Forward lunge  d) Drop jump | 0.334  -0.228  0.000  0.469  0.008  0.338  -0.030  0.423 |  | NA NA NA NA NA NA NA  NA | Inadequate |  |
|  | Side lying | 0.5°/s | Flex/ext | 20 |  | a) VPT  b) OLH  c) TAS  d) KOOS pain  e) KOOS symptoms  f) KOOS ADL  g) KOOS sport/rec  h) KOOS QoL  i) KOOS Q3  a) VPT  b) OLH  c) TAS  d) KOOS pain  e) KOOS symptoms  f) KOOS ADL  g) KOOS sport/rec  h) KOOS QoL  i) KOOS Q3 | -0.267, 0.162  -0.351  -0.208  -0.464  -0.046  -0.491  -0.212  -0.020  0.207  -0.190, -0.069  0.244  -0.137  0.160  0.075  0.192  0.040  0.121  -0.045 |  | -  -  -  -  -  -  -  -  -  -  -  -  -  -  -  -  -  - | Doubtful |  |
|  | Side lying | 1°/s | Flex/ext | 20 |  | a) Mini-squat  b) SLHD  c) COHD  a) Mini-squat  b) SLHD  c) COHD | 0.452  0.662  0.610  0.196  0.030  0.068 |  | -  -  -  -  -  - | Doubtful |  |
|  | Supine | 0.5°/s | Flex | 20 |  | a) Lysholm score  b) Tegner score: workload  c) Tegner score: athletics  d) Subjective assessment of performance  e) Retropatellar pain  f) Laxity at flexion 25°  g) Laxity at flexion 70°  h) Triple jump test  i) One-step leap test  j) Reproduction of an angle  a) Lysholm score  b) Tegner score: workload  c) Tegner score: athletics  d) Subjective assessment of performance  e) Retropatellar pain  f) Laxity at flexion 25°  g) Laxity at flexion 70°  h) Triple jump test  i) One-step leap test  j) Reproduction of an angle | NS  NS  NS  0.6  NS  NS  NS  NS  NS  0.6  NS  NS  NS  NS  NS  NS  NS  NS  NS  NS  NS  NS |  | -  -  -  -  -  -  -  -  -  -  -  -  -  -  -  -  -  -  -  -  -  - | Doubtful |  |
|  | Side lying | 0.5°/s | Flex/Ext | 20/40 |  | a) Age  b) Gender  c) Activity level  d) Associated meniscal  e) Chondral and collateral ligament lesions  f) Lachman  g) Pivot shift  h) Subjective ratings | NR  NR  NR  NR  NR  NR  NR  NR |  | ?  ? ? ? ?  ? ?  NA | Doubtful  Inadequate |  |
|  | Sitting | 0.5°/s | Flex | 45 |  | a) Tilt angle of dynamic balance | 0.579 |  | NA | Inadequate |  |
|  | Sitting | 0.5°/s | Flex/ext | 15/45 |  | Muscle strength  a) Extensors 60°/s  b) Flexors 60°/s  c) Extensors 180°/s  d) Flexors 180°/s  e) H/Q 60°/s  f) H/Q 180°/s | 0.34  0.33  0.45  0.30  0.54*  0.32 |  | -  -  -  -  -  - | Doubtful |  |
|  | Sitting | 1°/s | Flex/ext | 15 |  | a) Knee laxity KT-1000  a) Knee laxity KT-1000  a) Knee laxity KT-1000 | NR  NR  NR |  | ?  ?  ? | Inadequate |  |
|  | Sitting | 3°/s | Flex/ext | 15 |  | a) Knee laxity (KT-2000)  b) Lysholm score | NR  NR |  | ?  ? | Doubtful |  |
|  | Sitting | 0.5°/s | Flex/ext | 15 |  | KOOS  a) Pain (0–100)  b) Symptoms (0–100)  c) Activities of daily living (0–100)  d) Sport (0–100)  e) Quality of life (0–100)  f) Cincinnati knee score (0–100)  g) One-leg hop test  h) Stair hop test  i) KT-1000a | 0.21  0.17  0.09  0.14  0.33  0.21  0.40  0.15  0.03 |  | -  -  -  -  -  -  -  -  - | Doubtful |  |
|  | Side lying | 0.5°/s | Flex/ext | 20/40 |  | a) Single leg hop distance  b) Subjective function | -11.8314  -4.5180 |  | -  NA | Doubtful  Inadequate |  |
|  | Side lying | NR | Flex/ext | 20/40 |  | a) Age  b) Tegner activity score before  c) Tegner activity score after  d) Subjective function  e) Lachman  f) Lateral cartilage injury  g) Lateral meniscus injury  h) Medial cartilage injury  i) Medial meniscus injury  j) Medial collateral lig. injury  k) Gender | 0.249  0.002  -0.260  -0.357  0.330  0.307  0.009  0.056  0.085  -0.192  0.013 |  | -  -  -  -  -  -  -  -  -  -  - | Doubtful |  |
|  | Sitting | NR | Flex | 40 |  | a) Somatosensory evoked potentials (SEPs) | NR |  |  | Inadequate |  |
|  | Sitting | ca 0.5°/s | Flex/ext | 90 |  | a) Step length forward gait at different velocities of treadmill walking  b) Step length backward gait at different velocities of treadmill walking  a) Step length forward gait at different velocities of treadmill walking  b) Step length backward gait at different velocities of treadmill walking | NR  NR  NR  NR |  | NA  NA  NA  NA | Inadequate |  |
|  |  |  |  |  |  |  |  |  | **4+/95-/13?/16NA** | |  |
|  |  |  |  |  |  |  |  |  | **Insufficient** | |  |
|  |  |  |  |  |  |  |  |  | **Moderate** | |  |
| Abbreviations: ACLD = anterior cruciate ligament-deficient; ACLR = anterior cruciate ligament-reconstructed; ADL = Activities of Daily Living; COHD = Cross-Over Hop for Distance; Contra = contralateral; Ext = extension; Flex = flexion; H/Q = hamstring/quadriceps; KOOS = Knee injury and Osteoarthritis Outcome Score; KT-1000/2000 = knee arthrometer; Meth. = methodological; NR = not reported; OLH = One Leg Hop; PMP = psychometric property; QoL = Quality of Life; SA = starting angle; SLHD = Single Leg Hop for Distance; Sport/rec = sport and recreation function; TAS = Tegner Activity Scale; TTDPM = threshold to detect passive motion; VPT = Vibratory Perception Test.  Ratings: “+” = sufficient; “-” = insufficient; “?” = indeterminate; NA = not applicable due to rating of inadequate PMP | | | | | | | | | | | |
