# Supplementary material for: Properties of tests for knee joint threshold to detect passive motion following anterior cruciate ligament injury: a systematic review and meta-analysis
Source: J Orthop Surg Res. 2022 Mar 4;17:134. doi: 10.1186/s13018-022-03033-4 (PMC8895768; doi:10.1186/s13018-022-03033-4)
Supplement: Supplementary file 6 — Additional file 6: Table S6. Known-groups validity. [file 13018_2022_3033_MOESM6_ESM.docx]

**SUPPLEMENTAL TABLE S6**

Known-groups validity

| **Study (year)** |  | **TTDPM test details** | | | | |  | **Group 1** | | **Group 2** | | | **Outcome** | | **Quality** | |
| --- | --- | --- | --- | --- | --- | --- | --- | --- | --- | --- | --- | --- | --- | --- | --- | --- |
|  | |  | **Position** | **Angular velocity** | **Direction** | **SA (°)** |  | **N** | **AE mean ± SD (°)** | **N** |  | **AE mean ± SD (°)** | **p value**  **(if < 0.05)** | **Favours** | **PMP** | **Meth.** |
| *1. ACLD vs. 2. CTRL* | | | |  |  |  |  |  |  |  |  |  |  |  |  |  |
| Arockiaraj et al. (2013) | |  | Sitting | 0.2°/s | Ext | 30  75 |  | 25 | 1.44 ± 0.30  0.90 ± 0.20 | 25 |  | 0.97 ± 0.20  0.70 ± 0.11 | <0.001  <0.001 | CTRL  CTRL | +  + | Doubtful |
| Bonfim et al. (2009) | |  | Supine | 0.5°/s | Flex  Ext | 15  45  15  45 |  | 28 | NR  NR  NR  NR | 28 |  | NR  NR  NR  NR | <0.01  <0.01  <0.01  <0.01 | Unclear  Unclear  Unclear  Unclear | NA NA NA NA | Inadequate |
| Fischer-Rasmussen et al. (2000) | |  | Supine | 0.5°/s | Flex | 20 |  | 20 | 1.52±0.71 | 20 |  | 1.28±0.63 | NS | None | NA | Inadequate |
| Fonseca et al. (2005) | |  | Sitting | 2°/s | Ext | 35 |  | 11 | 0.88 ± 0.14 | 11 |  | 1.13 ± 0.19 | NS | None | - | Very good |
| Fridén et al.  (1997) | |  | Side lying | 0.5°/s | Ext  Flex | 20 40  20  40 |  | 16 | 1.0 ± 0.28  1.06 ± 0.35  0.94 ± 0.35  0.81 ± 0.21 | 19 |  | 0.75 ± 0.14  0.81 ± 0.20  0.94 ± 0.07  0.56 ± 0.07  Average both legs | NS  NS  NS  NS | None  None  None  None | -  -  -  - | Doubtful |
| Fridén et al. (1996) | |  | Side lying | 0.5°/s | Flex  Ext | 20  40  20  40 |  | 20 | 3.38 ± 2.54  NR  3.36 ± 3.08  NR | 19 |  | 1.38 ± 0.68  0.94 ± 0.47  1.06 ± 0.47  1.5 ± 0.95 | NS  NS  0.01  NS | None  None  CTRL  None | NA  NA NA NA | Inadequate |
| MacDonald et al. (1996) | |  | Sitting | 0.5°/s | Flex/ext | 30/40 |  | 10 | 0.80 ± 0.29 | 6 |  | 0.75 ± 0.09 | NS | None | NA | Inadequate |
| Ozenci et al. (2007) | |  | Sitting | 1°/s | Flex/ext | 15 |  | 20 | 1.93 ± 0.42 | 20 |  | 1.03 ± 0.91 | <0.001 | CTRL | + | Doubtful |
| Pap et al. (1997) | |  | Sitting | 0.15 m/s | Flex/ext | 45 |  | 20 | 1.07 ± 0.30 | 20 |  | 1.15 ± 0.36 | NS | None | - | Doubtful |
| Pap et al. (1999) | |  | Sitting | 0.1°/s  0.15°/s  0.35°/s  0.6°/s  0.85°/s | Flex/ext | 45 |  | 20 | 0.89 ± 0.43  1.13 ± 0.49  1.17 ± 0.59  1.24 ± 0.41  1.06 ± 0.49 | 15 |  | 0.97 ± 0.33  1.06 ± 0.48  0.89 ± 0.39  0.86 ± 0.54  1.03 ± 0.49 | NR  NR  NR  NR  NR | None  None  None  None  None | NA NA NA NA NA | Inadequate |
| Roberts et al. (1999) | |  | Side lying | 0.5°/s | Ext  Flex  Ext  Flex | 20  40  20  40  20  40  20  40 |  | 17  20 | 3.63 ± 3.20  4.38 ± 4.04  3.38 ± 2.64  2.13 ± 1.53  1.63 ± 0.94  0.88 ± 0.40  1.13 ± 0.67  1.13 ± 0.67 | 19  19 |  | 1.06 ± 0.47  1.5 ± 0.95  1.38 ± 0.68  0.94 ± 0.47  1.06 ± 0.47  1.5 ± 0.95  1.38 ± 0.68  0.94 ± 0.47 | 0.01  NS  NS  NS  NS NS NS NS | CTRL  None  None  None  None  None  None  None | NA NA NA NA NA NA NA NA | Inadequate |
| Viggiano et al (2014) | |  | Sitting | 0.5°/s | Flex/ext | 90 |  | 15 | NR | 15 |  | NR | NR | None | NA | Inadequate |
| *Pooled results* | |  |  |  |  |  |  |  |  |  |  |  |  |  | **3+/ 5-/ 24NA** | |
| *Quality of PMP* | |  |  |  |  |  |  |  |  |  |  |  |  |  | **Indeterminate** | |
| *Level of evidence* | |  |  |  |  |  |  |  |  |  |  |  |  |  | **Strong** | |
| *1. ACLR vs. 2. CTRL* | | | |  |  |  |  |  |  |  |  |  |  |  |  |  |
| Bonfim et al. (2003) | |  | Supine | 0.5°/s | Flex  Ext | 0  15  30  45  60  0  15  30  45  60 |  | 10 | NR  NR  NR  NR  NR  NR  NR  NR  NR  NR | 10 |  | NR  NR  NR  NR  NR  NR  NR  NR  NR  NR | NS  NS  NS  NS  NS  NS  NS  NS  NS  NS | None  None  None  None  None  None  None  None  None  None | NA NA NA NA NA NA NA NA NA NA | Inadequate |
| Co et al. (1993) | |  | Sitting | 0.5°/s | Flex | 40 |  | 10 | 1.33 ± 0.76 | 10 |  | 1.84 ± 0.75 | 0.001 | CTRL | + | Adequate |
| Courtney et al. (2019) | |  | Sitting | 0.5°/s | Flex/ext | 45 |  | 20 | 4.8 ± 1.6 | 6 |  | 2.1 ± 0.7 | <0.001 | CTRL | + | Adequate |
| Fischer-Rasmussen et al. (2000) | |  | Supine | 0.5°/s | Flex | 20 |  | 18 | 1.43 ± 0.52 | 20 |  | 1.28 ± 0.63 | NS | None | - | Doubtful |
| Laboute et al. (2019) | |  | Sitting | 4°/s | Flex | 15 |  | 32  32 | 1.22 ± 0.53  0.91 ± 0.35 | 32  32 |  | 0.66 ± 0.19  0.66 ± 0.19 | <0.001  NS | CTRL  None | +  - | Very good |
| MacDonald et al. (1996) | |  | Sitting | 0.5°/s | Flex/ext | 30/40 |  | 8  8 | 0.83 ± 20  0.84 ± 0.17 | 6  6 |  | 0.75 ± 0.09  0.75 ± 0.09 | NS  NS | None  None | NA NA | Inadequate |
| Nagai et al. (2013) | |  | Sitting | 0.25°/s | Flex  Ext | 15  15 |  | 11 | 0.9 ± 0.5  1.1 ± 0.8 | 11 |  | 1.3 ± 0.8  1.2 ± 1.1 | NS  NS | None  None | -  - | Doubtful |
| Ozenci et al. (2007) | |  | Sitting | 1°/s | Flex/ext | 15 |  | 20  20 | 1.01 ± 0.16  0.96 ± 0.27 | 20  20 |  | 1.03 ± 0.91  1.03 ± 0.91 | NR  NR | None  None | -  - | Doubtful |
| Reider et al (2003) | |  | Sitting | 3°/s | Flex/ext | 15 |  | 26 | 1.59 (NR) | 26 |  | 1.52 (NR) | NS | None | - | Doubtful |
| Risberg et al. (1999) | |  | Sitting | 0.5°/s | Flex/ext | 15 |  | 20 | 1.06 ± 0.57 | 10 |  | 1.55 ± 0.92 | 0.05 | ACLR | + | Doubtful |
| Roberts et al. (2000) | |  | Side lying | 0.5°/s | Flex  Ext | 20  40  20  40 |  | 20 | 2.38 ± 1.20  1.63 ± 0.94  2.25 ± 1.34  1.88 ± 1.20 | 19 |  | 1.38 ± 0.68  0.94 ± 0.47  1.06 ± 0.47  1.5 ± 0.95 | 0.004  0.0008  0.0003  NS | CTRL  CTRL  CTRL  None | NA NA NA NA | Inadequate |
| Viggiano et al. (2014) | |  | Sitting | 0.5°/s | Flex | 90 |  | 15 | NR | 15 |  | NR | NS | None | NA | Inadequate |
| Zandiyeh et al. (2019) | |  | Sitting | 0.25°/s | Flex/ext | 15 |  | 19 | 0.3 ± 0.1 | 28 |  | NR | 0.002 | CTRL | + | Doubtful |
| *Pooled results* | |  |  |  |  |  |  |  |  |  |  |  |  |  | **5+/ 7-/ 17NA** | |
| *Quality of PMP* | |  |  |  |  |  |  |  |  |  |  |  |  |  | **Indeterminate** | |
| *Level of evidence* | |  |  |  |  |  |  |  |  |  |  |  |  |  | **Strong** | |
| *1. ACLD vs 2. ACLR* | | | |  |  |  |  |  |  |  |  |  |  |  |  |  |
| Cronström (2018) | |  | Side lying | 1°/s | Flex/ext | 20 |  | 38 | 1.77 ± 0.98 | 13 |  | 2.28 ± 1.18 | NS | None | NA | Inadequate |
| MacDonald et al. (1996) | |  | Sitting | 0.5°/s | Flex/ext | 30/40 |  | 10  10 | 0.80 ± 0.29  0.80 ± 0.29 | 8  8 |  | 0.83 ± 0.20  0.84 ± 0.17 | NS  NS | None  None | NA NA | Inadequate |
| Ozenci et al. (2007) | |  | Sitting | 1°/s | Flex/ext | 15 |  | 20  20 | 1.93 ± 0.42  1.93 ± 0.42 | 20  20 |  | 1.01 ± 0.16  0.96 ± 0.27 | <0.001  NS | ACLR  None | +  - | Doubtful |
| Viggiano et al. (2014) | |  | Sitting | 0.5°/s | Flex/ext | 90 |  | 15 | NR | 15 |  | NR | NS | None | NA | Inadequate |
| *Pooled results* | |  |  |  |  |  |  |  |  |  |  |  |  |  | **1+/1-/4NA** | |
| *Quality of PMP* | |  |  |  |  |  |  |  |  |  |  |  |  |  | **Indeterminate** | |
| *Level of evidence* | |  |  |  |  |  |  |  |  |  |  |  |  |  | **Limited** | |
| *1. ACLR vs 2. ACLR* | | | |  |  |  |  |  |  |  |  |  |  |  |  |  |
| Angoules et al. (2011) | |  | Sitting | 2°/s | Flex  Ext | 15  45  15  45 |  | 20 | 1.25 ± 0.74  1.57 ± 0.57  1.15 ± 0.64  1.35 ± 0.61 | 20 |  | 1.15 ± 0.50  1.67 ± 0.59  1.30 ± 0.62  1.43 ± 0.59 | NS  NS  NS  NS | None  None  None  None | NA NA NA NA | Inadequate |
| Laboute et al. (2019) | |  | Sitting | 4°/s | Flex | 15 |  | 32 | 1.22 ± 0.53 | 32 |  | 0.91 ± 0.35 | <0.001 | ACLR (re-training) | + | Very good |
| Lee et al. (2008) | |  | NR | 0.5°/s | Ext | 15  30  45 |  | 9 | 1.54 ± 0.19  1.79 ± 0.13  1.89 ± 0.21 | 7 |  | 1.91 ± 0.19  2.18 ± 0.08  1.74 ± 0.13 | NS  0.031  NS | None  ACLR (group I)  None | NA  NA NA | Inadequate |
| Lephart et al. (1992) | |  | Sitting | 0.5°/s | Flex/ext | 15  45 |  | 6 | NR | 6 |  | NR | NS | None | NA | Inadequate |
| Ma et al. (2014) | |  | Sitting | 0.2°/s | Flex  Ext | 45 |  | 20  21 | 4.2 ± 2.0  7.4 ± 5.9  NR  NR | 26  26 |  | 3.2 ± 2.4  5.1 ± 3.8  3.2 ± 2.4  5.1 ± 3.8 | <0.05  NS  NS  NS | DB-group  None  None  None | +  -  -  - | Doubtful |
| MacDonald et al. (1996) | |  | Sitting | 0.5°/s | Flex/ext | 30/40 |  | 8 | 0.83 ± 0.20 | 8 |  | 0.84 ± 0.17 | NS | None | NA | Inadequate |
| Nakamae et al. (2014) | |  | Sitting | 0.2°/s | Flex/ext | 15/45 |  | 61  61  82 | NR  NR  NR | 82  73  73 |  | NR  NR  NR | NS  NS  NS | None  None  None | -  -  - | Doubtful |
| Ozenci et al. (2007) | |  | Sitting | 1°/s | Flex/ext | 15 |  | 20 | 1.01 ± 0.16 | 20 |  | 0.96 ± 0.27 | <0.01 | ACLR (Allograft) | + | Doubtful |
| Reider et al. (2003) | |  | Sitting | 3°/s | Flex/ext | 15 |  | 13 | NR | 13 |  | NR | NS | None | - | Doubtful |
| Risberg et al. (2007) | |  | NR | 0.5°/s | Flex/ext | 15 |  | 34 | 1.20 ± 0.76 | 31 |  | 1.22 ± 0.52 | NR | NR | ? | Doubtful |
| *Pooled results* | |  |  |  |  |  |  |  |  |  |  |  |  |  | **3+/7-/1?/8NA** | |
| *Quality of PMP* | |  |  |  |  |  |  |  |  |  |  |  |  |  | **Indeterminate** | |
| *Level of evidence* | |  |  |  |  |  |  |  |  |  |  |  |  |  | **Strong** | |
| *1. ACLD vs 2. ACLD* | | | |  |  |  |  |  |  |  |  |  |  |  |  |  |
| Courtney et al. (2005) | |  | Sitting | 0.5°/s | Flex/ext | 40 |  | 10  10  3 | 0.54 ± 0.11  0.54 ± 0.11  1.46 ± 0.43 | 3  4  4 |  | 1.46 ± 0.43  1.53 ± 0.61  1.53 ± 0.61 | NR  NR  NR | Unclear  Unclear  Unclear | NA NA  NA | Inadequate |
| Jensen et. al (2002) | |  | Supine | 0.5°/s | Ext | 20 |  | 7 | 1.23 ± 0.40 | 7 |  | 1.33 ± 0.48 | NS | None | NA | Inadequate |
| Roberts et al. (1999) | |  | Side lying | 0.5°/s | Ext  Flex | 20  40  20  40 |  | 17 | 3.63 ± 3.20  4.38 ± 4.04  3.38 ± 2.64  2.13 ± 1.53 | 20 |  | 1.63 ± 0.94  0.88 ± 0.40  1.13 ± 0.67  1.13 ± 0.67 | NS  0.014  0.012  NS | None  ACLD (group 2)  ACLD (group 2)  None | NA NA NA  NA | Inadequate |
| *Pooled results* | |  |  |  |  |  |  |  |  |  |  |  |  |  | **8NA** | |
| *Quality of PMP* | |  |  |  |  |  |  |  |  |  |  |  |  |  | **Not estimable** | |
| *Level of evidence* | |  |  |  |  |  |  |  |  |  |  |  |  |  | **Unknown** | |
| *1. ACL-injury vs 2. ACL- injury* | | | |  |  |  |  |  |  |  |  |  |  |  |  |  |
| Cronström et al. (2014) | |  | Side lying | 0.5°/s | Flex/ext | 20 |  | 28 men | 1.91 ± 0.87 | 23 wo-men |  | 2.43 ± 1.38 | NS | None | NA | Inadequate |
| *Pooled results* | |  |  |  |  |  |  |  |  |  |  |  |  |  | **1NA** | |
| *Quality of PMP* | |  |  |  |  |  |  |  |  |  |  |  |  |  | **Not estimable** | |
| *Level of evidence* | |  |  |  |  |  |  |  |  |  |  |  |  |  | **Unknown** | |
| *Total* | |  |  |  |  |  |  |  |  |  |  |  |  |  | **12+/20-/1?/62NA** | |
| *Quality of PMP* | |  |  |  |  |  |  |  |  |  |  |  |  |  | **Indeterminate** | |
| *Level of evidence* | |  |  |  |  |  |  |  |  |  |  |  |  |  | **Strong** | |
