# Supplementary material for: Properties of tests for knee joint threshold to detect passive motion following anterior cruciate ligament injury: a systematic review and meta-analysis
Source: J Orthop Surg Res. 2022 Mar 4;17:134. doi: 10.1186/s13018-022-03033-4 (PMC8895768; doi:10.1186/s13018-022-03033-4)
Supplement: Supplementary file 7 — Additional file 7: Table S7. Discriminative validity. [file 13018_2022_3033_MOESM7_ESM.docx]

**SUPPLEMENTAL TABLE S7**

Discriminative validity

| **Study (year)** | **TTDPM test details** | | | | **N** | **Injured knee** | **Contralateral knee** | **Outcome** | | **Quality** | |  |
| --- | --- | --- | --- | --- | --- | --- | --- | --- | --- | --- | --- | --- |
|  | **Position** | **Angular velocity** | **Direction** | **SA (°)** |  | **AE mean ± SD (°)** | **AE mean ± SD (°)** | **p value**  **(if < 0.05)** | **Favours** | **PMP** | **Meth.** |  |
| *ACLD* |  |  |  |  |  |  |  |  |  |  |  |  |
| Arockiaraj et al. (2013) | Sitting | 0.2°/s | Ext | 30  75 | 25 | 1.44 ± 0.30  0.90 ± 0.20 | 0.87 ± 0.19  0.78 ± 0.19 | <0.001  0.035 | Contra  Contra | NA  NA | Inadequate |  |
| Barrack et al. (1989) | Sitting | 0.5°/s | Ext | 40 | 11 | 3.53 ± 1.22 | 2.57 ± 0.59 | 0.008 | Contra | NA | Inadequate |  |
| Beynnon et al. (1999) | Sitting | 0.1°/s | Flex/ext | 45° | 20 | 1.45 ± 0.50 | 1.17 ± 0.50 | 0.011 | Contra | + | Doubtful |  |
| Borsa et al. (1997) | Sitting | 0.5°/s | Flex/ext | 15/45 | 29 | NR | NR | <0.01 | Unclear | ? | Inadequate |  |
| Courtney et al. (2005) | Sitting | 0.5°/s | Flex/ext | 40 | 10  3  4 | 0.54 ± 0.11  1.46 ± 0.43  1.53 ± 0.61 | 0.54 ± 0.12  0.68 ± 0.24  0.79 ± 0.27 | NR  NR  NR | Unclear  Unclear  Unclear | ?  ?  ? | Inadequate |  |
| Fischer-Rasmussen et al. (2000) | Supine | 0.5°/s | Flex | 20 | 20 | 1.52 ± 0.71 | 1.31 ± 0.63 | 0.01 | Contra | + | Doubtful |  |
| Fischer-Rasmussen et al. (2001) | Supine | 0.5°/s | Flex | 20 | 10 | 1.48 ± 0.81 | 1.44 ± 0.65 | NR | Unclear | ? | Inadequate |  |
| Fonseca et al. (2005) | Sitting | 2°/s | Ext | 35 | 11 | 0.88 ± 0.14 | 0.87 ± 0.13 | NS | None | - | Very good |  |
| Fridén et al.  (1997) | Side lying | 0.5°/s | Ext  Flex | 20  40  20  40 | 16 | 1.0 ± 0.28  1.06 ± 0.35  0.94 ± 0.35  0.81 ± 0.21 | 0.69 ± 0.21  0.81 ± 0.35  1.0 ± 0.28  0.43 ± 0.14 | NR  NR  NR  NR | Unclear  Unclear  Unclear  Unclear | ? ? ? ? | Doubtful |  |
| Fridén et al. (1996) | Side lying | 0.5°/s | Flex  Ext | 20  40  20  40 | 20 | NR  NR  NR  NR | NR  NR  NR  NR | NS  NS  NS  NS | None  None  None  None | NA NA NA NA | Inadequate |  |
| Jensen et al. (2002) | Supine | 0.5°/s | Ext | 20 | 7  7 | 1.23 ± 0.40  1.33 ± 0.48 | 1.18 ± 0.40  1.43 ± 0.55 | NS  NS | None  None | NA NA | Inadequate |  |
| Lee et al. (2009) | Sitting | 0.5°/s | Flex | 45 | 12 | 3.76 ± 2.60 | 2.61 ± 1.95 | 0.015 | Contra | NA | Doubtful |  |
| MacDonald et al. (1996) | Sitting | 0.5°/s | Flex/ext | 30/40 | 10 | 0.80 ± 0.29 | 0.68 ± 0.22 | 0.0041 | Contra | NA | Inadequate |  |
| Ozenci et al. (2007) | Sitting | 1°/s | Flex/ext | 15 | 20 | 1.93 ± 0.42 | 1.87 ± 0.38 | 0.001 | Contra | + | Doubtful |  |
| Pap et al. (1997) | Sitting | 0.15 m/s | Flex/ext | 45 | 20 | 1.07 ± 0.30 | 1.12 ± 0.31 | NS | None | - | Doubtful |  |
| Pap et al. (1999) | Sitting | 0.1°/s  0.15°/s  0.35°/s  0.6°/s  0.85°/s | Flex/ext | 45 | 20 | 0.89 ± 0.43  1.13 ± 0.49  1.17 ± 0.59  1.24 ± 0.41  1.06 ± 0.49 | 1.21 ± 0.49  1.06 ± 0.55  0.99 ± 0.57  1.06 ± 0.35  1.19 ± 0.47 | NS  NS  NS  NS  NS | None  None  None  None  None | -  -  -  -  - | Doubtful |  |
| Valeriani et al. (1996) | Sitting | NR | Flex | 40 | 19 | NR | NR | NR | None | NA | Inadequate |  |
| *Pooled results* |  |  |  |  |  |  |  |  |  | **3+/7-/9?/12NA** | |  |
| *Quality of PMP* |  |  |  |  |  |  |  |  |  | **Indeterminate** | |  |
| *Level of evidence* |  |  |  |  |  |  |  |  |  | **Strong** | |  |
| *ACLR* |  |  |  |  |  |  |  |  |  |  |  |  |
| Angoules et al. (2011) | Sitting | 2°/s | Flex  Ext  Flex  Ext | 15  45  15  45  15  45  15  45 | 20  20 | 1.25 ± 0.74  1.57 ± 0.57  1.15 ± 0.64  1.35 ± 0.61  1.15 ± 0.50  1.67 ± 0.59  1.30 ± 0.62  1.43 ± 0.59 | 1.16 ± 1.02  1.40 ± 0.82  1.40 ± 1.00  1.42 ± 0.84  0.87 ± 0.49  1.38 ± 0.77  1.33 ± 0.83  1.57 ± 0.82 | NS  NS  NS  NS  0.006  NS  NS  NS | None  None  None  None  Contra  None  None  None | NA NA NA NA NA NA NA NA | Inadequate |  |
| Co et al. (1993) | Sitting | 0.5°/s | Flex | 40 | 10 | 1.33 ± 0.76 | 1.20 ± 0.41 | 0.05 | None | - | Adequate |  |
| Courtney et al. (2019) | Sitting | 0.5°/s | Flex/ext | 45 | 20 | 4.8±1.6 | 3.5±1.3 | <0.001 | Contra | + | Adequate |  |
| Fischer-Rasmussen et al. (2000) | Supine | 0.5°/s | Flex | 20 | 18 | 1.43 ± 0.52 | 1.26 ± 0.49 | NS | None | - | Doubtful |  |
| Gupta RK et al. (2010) | NR | NR | NR | NR | 45 | 1.359 (NR) | 1.247 (NR) | <0.01 | Contra | NA | Inadequate |  |
| Laboute et al. (2019) | Sitting | 4°/s | Flex | 15 | 32  32 | 1.22 ± 0.53  0.91 ± 0.35 | 0.95 ± 0.29  0.86 ± 0.34 | 0.001  NS | Contra  None | +  - | Very good |  |
| Lee et al. (2008) | NR | 0.5°/s | Ext | 15/30/45 | 9  7 | NR  NR | NR  NR | NS  NS | None  None | -  - | Inadequate |  |
| Lephart et al. (1992) | Sitting | 0.5°/s | Ext  Flex | 15  45  15  45 | 12 | NR  NR  NR  NR | NR  NR  NR  NR | <0.01  NS  <0.05  NS | Unclear  None  Unclear  None | NA NA NA NA | Inadequate |  |
| MacDonald et al. (1996) | Sitting | 0.5°/s | Flex/ext | 30/40 | 8  8 | 0.83 ± 0.20  0.84 ± 0.17 | 0.68 ± 0.16  0.62 ± 0.18 | 0.0027  0.0001 | Contra  Contra | NA  NA | Inadequate |  |
| Nagai et al. (2013) | Sitting | 0.25°/s | Flex  Ext | 15  15 | 11 | 0.9 ± 0.5  1.1 ± 0.8 | 1.0 ± 0.6  1.1 ± 0.9 | NS  NS | None  None | -  - | Doubtful |  |
| Nishiwaki et al. (2007) | Sitting | 0.5°/s | Flex  Ext | 15  45  15  45 | 16 | 0.9 ± 0.5  0.8 ± 0.4  0.8 ± 0.2  0.9 ± 0.6 | 0.9 ± 0.6  0.8 ± 0.2  0.9 ± 0.3  0.9 ± 0.9 | NR  NR  NR  NR | None  None  None  None | -  -  -  - | Doubtful |  |
| Ozenci et al. (2007) | Sitting | 1°/s | Flex/ext | 15 | 20  20 | 1.01 ± 0.16  0.96 ± 0.27 | 1.01 ± 0.16  1.12 ± 0.28 | 0.001  0.001 | None  Contra | -  + | Doubtful |  |
| Reider et al. (2003) | Sitting | 3°/s | Flex/ext | 15 | 26 | 1.59 (NR) | 1.46 (NR) | NS | None | - | Doubtful |  |
| Risberg et al. (1999) | Sitting | 0.5°/s | Flex/ext | 15 | 20 | 1.06 ± 0.57 | 1.00 ± 0.49 | NS | None | - | Inadequate |  |
| Roberts D et al. (2000) | Side lying | 0.5°/s | Flex  Ext | 20  40  20  40 | 20 | 2.38 ± 1.20  1.63 ± 0.94  2.25 ± 1.34  1.88 ± 1.20 | 1.13 ± 0.40  2.13 ± 0.94  1.13 ± 0.40  1.38 ± 0.67 | NS  NS  0.0507  NS | None  None  None  None | -  -  -  - | Doubtful |  |
| Shidahara et al. (2011) | Sitting | 0.2°/s  0.2°/s  0.2°/s  0.2°/s  0.1°/s  0.1°/s 0.1°/s  0.1°/s | Ext  Flex  Ext  Flex  Ext  Flex  Ext  Flex | 45  45  15  15  45  45  15  15 | 31 | 7.5 ± 1.3  5.4 ± 1.3  NR  NR  NR  NR  NR  NR | 5.1 ± 0.8  2.5 ± 0.6  NR  NR  NR  NR  NR  NR | 0.034  0.0033  NS  NS  NS  NS  NS  NS | Contra  Contra  None  None  None  None  None  None | +  +  -  -  -  -  -  - | Doubtful |  |
| *Pooled results* |  |  |  |  |  |  |  |  |  | **5+/24-/15NA** | |  |
| *Quality of PMP* |  |  |  |  |  |  |  |  |  | **Insufficient** | |  |
| *Level of evidence* |  |  |  |  |  |  |  |  |  | **Strong** | |  |
| *Total* |  |  |  |  |  |  |  |  |  | **8+/31-/9?/27NA** | |  |
| *Quality of PMP* |  |  |  |  |  |  |  |  |  | **Insufficient** | |  |
| *Level of evidence* |  |  |  |  |  |  |  |  |  | **Strong** | |  |
| Abbreviations: ACLD = anterior cruciate ligament-deficient; ACLR = anterior cruciate ligament-reconstructed; Contra = contralateral; CTRL = control group; Ext = extension; Fig. = figure; Flex = flexion; Meth. = methodological; NR = not reported; NS = not significant; PMP = psychometric property; SA = starting angle; TTDPM = threshold to detect passive motion.  Ratings: “+” = sufficient; “-” = insufficient; “?” = indeterminate “NA” = not applicable due to rating of inadequate PMP | | | | | | | | | | | | |
