# Supplementary material for: Properties of tests for knee joint threshold to detect passive motion following anterior cruciate ligament injury: a systematic review and meta-analysis
Source: J Orthop Surg Res. 2022 Mar 4;17:134. doi: 10.1186/s13018-022-03033-4 (PMC8895768; doi:10.1186/s13018-022-03033-4)
Supplement: Supplementary file 8 — Additional file 8: Table S8. Responsiveness between subgroups. [file 13018_2022_3033_MOESM8_ESM.docx]

**SUPPLEMENTAL TABLE S8**

Responsiveness between subgroups

| **Study (year)** | **TTDPM test details** | | | |  | **Populations** | | |  | **Outcome** | |  | **Quality** | |
| --- | --- | --- | --- | --- | --- | --- | --- | --- | --- | --- | --- | --- | --- | --- |
|  | **Position** | **Angular velocity** | **Direction** | **SA (°)** |  | **ACL/**  **CTRL** | **N** | **Intervention** |  | **p value**  **(if < 0.05)** | **Favours** |  | **PMP** | **Meth.** |
| Ageberg et al. (2012) | Side lying | NR | Flex/ext | 20 |  | ACLD  ACLR | 15  24 | Local anesthetic cream EMLA or placebo cream  Local anesthetic cream EMLA or placebo cream |  | NS  NS | None  None |  | NA NA | Inadequate |
| Angoules et al. (2011) | Sitting | 2°/s | Flex/ext | 15/45 |  | ACLR  ACLR | 20  20 | Hamstring (semitendinosus/gracilis) autograft  Bone–patellar tendon–bone autograft (BPB) |  | NS  NS | None  None |  | NA NA | Inadequate |
| Bonfim et al. (2009) | Supine | 0.5°/s | Flex/ext | 15/45 |  | ACLD | 28 | Before and after applying infrapatellar tape/strap |  | <0.01 | Intervention |  | NA | Inadequate |
| Ma et al. (2014) | Sitting | 0.2°/s | Flex/ext | 45 |  | ACLR  ACLR  ACLR | 20  21  26 | Before and after single-bundle (SB) reconstruction  Before and after single-bundle augmentation (SBA) reconstruction  Before and after double-bundle (DB) reconstruction |  | NS  NS  <0.05 | None  None  DB |  | NA  NA  NA | Inadequate |
| Risberg et al. (2007) | NR | 0.5°/s | Flex/ext | 15 |  | ACLR  ACLR | 39  35 | Patellar tendon op and NT rehabilitation program  Patellar tendon op and ST rehabilitation program |  | NS  NS | None  none |  | -  - | Doubtful |
| Shen et al. (2019) | Supine | 0.1°/s | Flex/ext | 20/50/80 |  | ACLR  ACLR  ACLR  ACLR  ACLR | 10  11  11  10  10 | Backward walking, 0° incline  Backward walking, 5° incline  Backward walking, 10° incline  Backward walking, 15° incline  Standard rehabilitation |  | NR  NR  NR  NR  NR | Unclear  Unclear  Unclear  Unclear  Unclear |  | ? ? ? ? ? | Doubtful |
| Zandiyeh et al. (2019) | Sitting | 0.25°/s | Flex/ext | 15 |  | ACLR  CTRL | 19  28 | With and without Stochastic Resonance (SR)  With and without Stochastic Resonance (SR) |  | 0.012  NS | SR  None |  | +  - | Doubtful |
| *Pooled results* |  |  |  |  |  |  |  |  |  |  |  |  | **1+/3-/5?/8NA** | |
| *Quality of PMP* |  |  |  |  |  |  |  |  |  |  |  |  | **Insufficient** | |
| *Level of evidence* | | |  |  |  |  |  |  |  |  |  |  | **Moderate** | |
| Abbreviations: ACLD = anterior cruciate ligament-deficient; ACLR = anterior cruciate ligament-reconstructed; Contra = contralateral; CTRL = control group; Ext = extension; Fig. = figure; Flex = flexion; Meth. = methodological; NR = not reported; NS = not significant; PMP = psychometric property; SA = starting angle; TTDPM = threshold to detect passive motion.  Ratings: “+” = sufficient; “-” = insufficient; “?” = indeterminate “NA” = not applicable due to rating of inadequate PMP | | | | | | | | | | | | | | |
