# Supplementary material for: Properties of tests for knee joint threshold to detect passive motion following anterior cruciate ligament injury: a systematic review and meta-analysis
Source: J Orthop Surg Res. 2022 Mar 4;17:134. doi: 10.1186/s13018-022-03033-4 (PMC8895768; doi:10.1186/s13018-022-03033-4)
Supplement: Supplementary file 9 — Additional file 9: Table S9. Responsiveness to intervention. [file 13018_2022_3033_MOESM9_ESM.docx]

**SUPPLEMENTAL TABLE S9**

Responsiveness to intervention

| **Study (year)** | **Population** | |  | **TTDPM test details** | | | |  | **Intervention** | **Outcome** | |  | **Quality** | |
| --- | --- | --- | --- | --- | --- | --- | --- | --- | --- | --- | --- | --- | --- | --- |
|  | **ACLD/**  **ACLR** | **n** |  | **Position** | **Angular velocity (°/s)** | **Direction** | **SA (°)** |  |  | **p value (if sig.)** | **Favours** |  | **PMP** | **Meth.** |
| Beynnon et al. (1999) | ACLD | 12 |  | Sitting | 0.5 | Flex | 45 |  | Knee brace  Neoprene sleeve | NS | None |  | - | Doubtful |
| Beynnon et al. (2011) | ACLR  ACLR | 19  17 |  | Sitting | 0.1 | Flex/ext | NR |  | Accelerated rehabilitation  Nonaccelerated rehabilitation | NS  NS | None  None |  | -  - | Very good |
| Gupta et al. (2010) | ACLR | 45 |  | NR | NR | NR | NR |  | Quadrupled STG graft with preserved tibial insertions | ≤ 0.01 | Intervention |  | NA | Inadequate |
| Lephart et al. (1992) | ACLR | 12 |  | Sitting | 0.5 | Ext  Flex | 15  45  15  45 |  | Neoprene sleeve | NS  <0.05  NS  <0.01 | None  Intervention  None  Intervention |  | NA  NA NA NA | Inadequate |
| Risberg et al. (1999) | ACLR | 20 |  | Sitting | 0.5 | Flex/ext | 15 |  | Knee brace and neoprene sleeve | NS | None |  | - | Adequate |
| Roberts et al. (2004) | ACLD | 36 |  | Side lying | 0.5 | Ext  Flex | 20  40  20  40 |  | 5-8 min cycle | NS  NS  0.034  NS | None  None  Intervention  None |  | NA NA NA NA | Inadequate |
| Shen et al. (2019) | ACLR  ACLR  ACLR  ACLR  ACLR | 10  10  10  11  11  11  11  11  11  10  10  10  10  10  10 |  | Supine | 1 | Flex/ext | 20  50  80  20  50  80  20  50  80  20  50  80  20  50  80 |  | Backward walking, 0° incline  Backward walking, 5° incline  Backward walking, 10° incline  Backward walking, 15° incline  Standard rehabilitation | <0.01  <0.01  <0.01  <0.001  <0.001  <0.001  <0.001  <0.001  <0.001  <0.01  <0.01  <0.01  NS  NS  NS | Intervention  Intervention  Intervention  Intervention  Intervention  Intervention  Intervention  Intervention  Intervention  Intervention  Intervention  Intervention  None  None  None |  | NA NA  NA  NA  NA  NA  NA  NA  NA  NA  NA  NA  NA  NA  NA | Inadequate |
| Shidahara et al. (2011) | ACLR | 31 |  | Sitting | 0.1  0.1  0.2  0.2 | Flex  Ext  Flex  Ext | 15  45  15  45  15  45  15  45 |  | ACLR with rehabilitation | NS  NS  NS  NS  NS  0.0033  NS  0.034 | None  None  None  None  None  Intervention  None  Intervention |  | -  -  -  -  -  +  -  + | Doubtful |
| Valeriani et al. (1999) | ACLR | 7 |  | Sitting | NR | Ext | 40 |  | Patella tendon autograft | NS | None |  | NA | Inadequate |
| Zandiyeh et al. (2019) | ACLR | 19 |  | Sitting | 0.25 | Flex/ext | 15 |  | Stochastic resonance | NS | None |  | - | Doubtful |
| *Pooled results* | |  |  |  |  |  |  |  |  |  |  | **2 +/ 11 -/ 25 NA** | | |
| *Quality of PMP* | |  |  |  |  |  |  |  |  |  |  | **Insufficient** | | |
| *Level of evidence* | |  |  |  |  |  |  |  |  |  |  | **Strong** | | |
| Abbreviations: TTDPM = threshold to detect passive motion; ACLD = anterior cruciate ligament-deficient; ACLR = anterior cruciate ligament-reconstructed; SA = starting angle; Flex = flexion; Ext = extension; NR = not reported; PMP = psychometric property; Meth. = methodological; STG = semitendinosus-gracilis; NA = not applicable | | | | | | | | | | | | | | |
